# Counteracting lineage-specific transcription factor network finely tunes lung adeno-to-squamous transdifferentiation through remodeling tumor immune microenvironment

**DOI:** 10.1093/nsr/nwad028

**Published:** 2023-02-14

**Authors:** Shijie Tang, Yun Xue, Zhen Qin, Zhaoyuan Fang, Yihua Sun, Chongzhe Yuan, Yunjian Pan, Yue Zhao, Xinyuan Tong, Jian Zhang, Hsinyi Huang, Yuting Chen, Liang Hu, Dasong Huang, Ruiqi Wang, Weiguo Zou, Yuan Li, Roman K Thomas, Andrea Ventura, Kwok-Kin Wong, Haiquan Chen, Luonan Chen, Hongbin Ji

**Affiliations:** State Key Laboratory of Cell Biology, Shanghai Institute of Biochemistry and Cell Biology, Center for Excellence in Molecular Cell Science, Chinese Academy of Sciences, Shanghai 200031, China; State Key Laboratory of Cell Biology, Shanghai Institute of Biochemistry and Cell Biology, Center for Excellence in Molecular Cell Science, Chinese Academy of Sciences, Shanghai 200031, China; College of Life Sciences, University of Chinese Academy of Sciences, Beijing 100049, China; State Key Laboratory of Cell Biology, Shanghai Institute of Biochemistry and Cell Biology, Center for Excellence in Molecular Cell Science, Chinese Academy of Sciences, Shanghai 200031, China; State Key Laboratory of Cell Biology, Shanghai Institute of Biochemistry and Cell Biology, Center for Excellence in Molecular Cell Science, Chinese Academy of Sciences, Shanghai 200031, China; Zhejiang University-University of Edinburgh Institute, Zhejiang University School of Medicine, Haining 314400, China; The Second Affiliated Hospital, Zhejiang University School of Medicine, Hangzhou 310000, China; Department of Thoracic Surgery, Fudan University Shanghai Cancer Center, Shanghai 200032, China; Department of Oncology, Shanghai Medical College, Fudan University, Shanghai 200032, China; Department of Thoracic Surgery, Fudan University Shanghai Cancer Center, Shanghai 200032, China; Department of Oncology, Shanghai Medical College, Fudan University, Shanghai 200032, China; Department of Thoracic Surgery, Fudan University Shanghai Cancer Center, Shanghai 200032, China; Department of Oncology, Shanghai Medical College, Fudan University, Shanghai 200032, China; Department of Thoracic Surgery, Fudan University Shanghai Cancer Center, Shanghai 200032, China; Department of Oncology, Shanghai Medical College, Fudan University, Shanghai 200032, China; State Key Laboratory of Cell Biology, Shanghai Institute of Biochemistry and Cell Biology, Center for Excellence in Molecular Cell Science, Chinese Academy of Sciences, Shanghai 200031, China; State Key Laboratory of Cell Biology, Shanghai Institute of Biochemistry and Cell Biology, Center for Excellence in Molecular Cell Science, Chinese Academy of Sciences, Shanghai 200031, China; State Key Laboratory of Cell Biology, Shanghai Institute of Biochemistry and Cell Biology, Center for Excellence in Molecular Cell Science, Chinese Academy of Sciences, Shanghai 200031, China; State Key Laboratory of Cell Biology, Shanghai Institute of Biochemistry and Cell Biology, Center for Excellence in Molecular Cell Science, Chinese Academy of Sciences, Shanghai 200031, China; School of Life Science and Technology, Shanghai Tech University, Shanghai 200120, China; State Key Laboratory of Cell Biology, Shanghai Institute of Biochemistry and Cell Biology, Center for Excellence in Molecular Cell Science, Chinese Academy of Sciences, Shanghai 200031, China; Department of Mathematics, Shanghai University, Shanghai 200444, China; Department of Mathematics, Shanghai University, Shanghai 200444, China; State Key Laboratory of Cell Biology, Shanghai Institute of Biochemistry and Cell Biology, Center for Excellence in Molecular Cell Science, Chinese Academy of Sciences, Shanghai 200031, China; Department of Thoracic Surgery, Fudan University Shanghai Cancer Center, Shanghai 200032, China; Department of Oncology, Shanghai Medical College, Fudan University, Shanghai 200032, China; Department of Translational Genomics, Center of Integrated Oncology Cologne-Bonn, Medical Faculty, University of Cologne, Cologne 50931, Germany; Department of Pathology, University Hospital Cologne, Cologne 50937, Germany; Cancer Biology and Genetics Program, Memorial Sloan Kettering Cancer Center, New York, NY 10065, USA; Laura and Isaac Perlmutter Cancer Center, New York University Langone Medical Center, New York, NY 10016, USA; Department of Thoracic Surgery, Fudan University Shanghai Cancer Center, Shanghai 200032, China; Department of Oncology, Shanghai Medical College, Fudan University, Shanghai 200032, China; State Key Laboratory of Cell Biology, Shanghai Institute of Biochemistry and Cell Biology, Center for Excellence in Molecular Cell Science, Chinese Academy of Sciences, Shanghai 200031, China; School of Life Science and Technology, Shanghai Tech University, Shanghai 200120, China; Key Laboratory of Systems Biology, Shanghai Institutes for Biological Sciences, Chinese Academy of Sciences, Shanghai 200031, China; State Key Laboratory of Cell Biology, Shanghai Institute of Biochemistry and Cell Biology, Center for Excellence in Molecular Cell Science, Chinese Academy of Sciences, Shanghai 200031, China; School of Life Science and Technology, Shanghai Tech University, Shanghai 200120, China; School of Life Science, Hangzhou Institute for Advanced Study, University of Chinese Academy of Sciences, Chinese Academy of Sciences, Hangzhou 310024, China

**Keywords:** LUAS, ALK fusion, counteracting TF regulatory network, CXCL3/CXCL5, LSD1

## Abstract

Human lung adenosquamous cell carcinoma (LUAS), containing both adenomatous and squamous pathologies, harbors strong plasticity and is significantly associated with poor prognosis. We established an up-to-date comprehensive genomic and transcriptomic landscape of LUAS in 109 Chinese specimens and demonstrated LUAS development via adeno-to-squamous transdifferentiation. Unsupervised transcriptomic clustering and dynamic network biomarker analysis identified an inflammatory subtype as the critical transition stage during LUAS development. Dynamic dysregulation of the counteracting lineage-specific transcription factors (TFs), containing adenomatous TFs NKX2-1 and FOXA2, and squamous TFs TP63 and SOX2, finely tuned the lineage transition via promoting CXCL3/5-mediated neutrophil infiltration. Genomic clustering identified the most malignant subtype featured with STK11-inactivation, and targeting LSD1 through genetic deletion or pharmacological inhibition almost eradicated STK11-deficient lung tumors. These data collectively uncover the comprehensive molecular landscape, oncogenic driver spectrum and therapeutic vulnerability of Chinese LUAS.

## INTRODUCTION

Human lung adenosquamous cell carcinoma (LUAS), accounting for ∼0.7%–11.4% of non-small-cell lung cancer (NSCLC), represents as a unique subtype with high malignancy and strong plasticity [1–5]. In contrast to human lung adenocarcinoma (LUAD) or lung squamous cell carcinoma (LUSC), LUAS with mixed adenomatous and squamous pathologies is highly resistant to conventional therapy [6,7]. LUAS patients tend to have worse prognoses than LUAD or LUSC patients [8]. Previous studies have shown that EGFR, TP53 and PI3KCA mutations and ALK fusion are frequently observed in human LUAS [9–13]. Analyses of micro-dissected specimens within single LUAS tumors show that most oncogenic mutations are shared between the adenomatous and squamous components [10,11,13], indicative of a potential link between these two pathologies. Two major hypotheses, tumor collision and lineage transition, have been proposed to explain the development of human LUAS [14]. The tumor collision hypothesis refers to the tangling growth of two different tumors whereas the lineage transition hypothesis favors pathological transformation within single tumors. Recent experimental evidence likely supports the lineage transition hypothesis based on the observation of identical genetic alterations shared between the adenomatous and squamous components [3,11,15,16]. Following this thread, LUAS is potentially derived from histological transformation during cancer malignant progression and/or drug resistance acquisition [6,17,18]. Indeed, multiple clinical studies have shown that squamous cancer arises from those LUAD patients relapsed from molecular targeted therapy [6,10,19,20], indicative of the potential contribution of lineage transition to drug resistance.

Great effort in recent years has established the comprehensive genomic and transcriptomic landscapes of lung cancer [21–30]. Molecular clustering analyses classify human LUAD into three major subtypes: proximal inflammatory (PI), proximal proliferative (PP) and terminal respiratory unit (TRU), whereas LUSC is mainly divided into four subtypes: classical, primitive, basal and secretory [27,28]. Moreover, large-scale protein mass spectrometry data also provide another layer of information for integrative lung cancer classification [31–34], i.e. the proteomic and transcriptomic analyses divide LUAD into another three subtypes: environment and metabolism high, mixed type and proliferation and proteasome group [33]. Studies have also established the proteomic landscape of LUSC, and identified new therapeutic targets and biomarkers for CDK4/6 inhibitor therapy [31]. In contrast to LUAD and LUSC, LUAS is more plastic and malignant but with a relatively low percentage, which makes sample collection and genomic analyses difficult. Despite previous efforts in small-scale studies, the systematic and comprehensive landscape of human LUAS is still lacking.

Multiple oncogenic drivers have been identified in human LUAS. *STK11/LKB1* (serine-threonine kinase 11) is mutated in ∼17% human LUAD [11] whereas its mutation rate is enriched in LUAS, averaging at 39.66% (ranging from 22% to 66% in multiple studies) [11,35–38], which is indicative of the potential role of STK11/LKB1 inactivation in LUAS development. Interestingly, we find that *Stk11/Lkb1* inactivation is able to drive AST in the *Kras^G12D^*-based genetically engineered mouse models (GEMMs) [35,39–41]. These pathologically transitioned tumors display therapeutic resistance to multiple inhibitors initially effective in LUAD [41]. A recent study has analyzed the STK11/LKB1 mutations in relapsed patients with potential adeno-to-squamous transdifferentiation, and shows a relatively low rate at 14.3% (1 out of 7) [11]. Such a low STK11 mutation rate could be ascribed to the mutual exclusivity between STK11 and EGFR mutations [42–44] since the majority of these patients have EGFR genetic alterations, e.g. 4/7 with EGFR mutations and 1/7 with EGFR amplification [11]. Excluding these EGFR-altered samples, the STK11/LKB1 mutation rate ranges from 33% to 50%, similar to previous reports [11,35–38]. Besides STK11, other drivers such as PI3K-AKT and MYC are also found to potentially regulate AST [11,39]. Systematic study of human LUAS genomics and transcriptomics might uncover the full spectrum of oncogenic drivers in this highly malignant NSCLC subtype.

We here analyzed 109 human LUAS specimens, the largest cohort to date, through RNA-sequencing (RNA-seq) and whole-genome sequencing (WGS) with the integration of laser-capture microdissection (LCM). We established the comprehensive transcriptomic and genomic landscape and oncogenic driver spectrum of human LUAS, and further identified its therapeutic vulnerability.

## RESULTS

### Clinical information for Chinese LUAS

**Figure 1.**
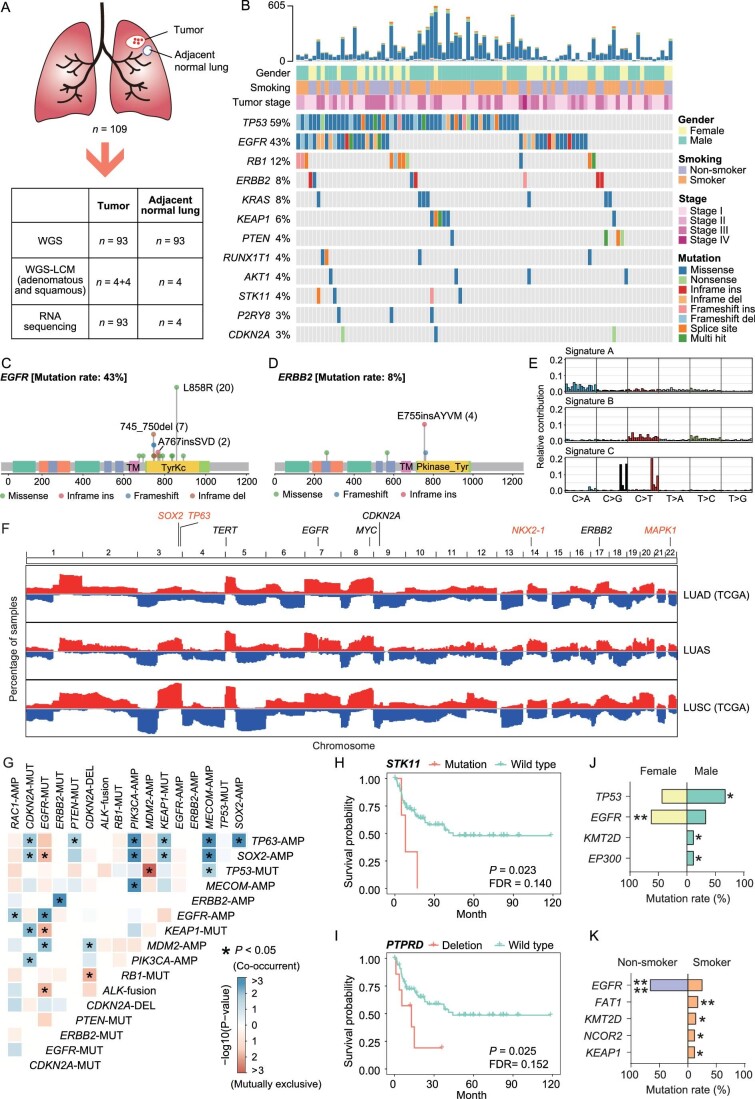
The genomic landscape of Chinese LUAS. (A) Brief description of Chinese LUAS genomics and transcriptomics study. (B) Somatic mutation plot of 93 LUAS samples. Significantly mutated cancer-related genes (*P* value < 0.1 and mutation rate ≥ 3%) reported in the OncoKB database and COSMIC database were ranked in order of decreasing prevalence. Mutation types are indicated and frequency of mutations is shown on the left. The top graph indicates total non-synonymous mutation numbers within coding regions across each sample, followed by clinical features involving gender, smoking status and stage. (C and D) Mutation types and hot-spot mutations of (C) *EGFR* and (D) *ERBB2*. (E) *De novo* mutational signatures identified in LUAS. (F) Copy number variation (CNV) segments of TCGA-LUAD (top), Chinese LUAS (middle) and TCGA-LUSC (bottom) displayed by Integrated Genomics Viewer (IGV). Amplification in red; deletion in blue. Cancer-related genes are marked on top. (G) Plot of concurrent and mutually exclusive events among top amplified or deleted cancer-related genes (alteration rate ≥ 5%) together with driver mutations. AMP/DEL/MUT represent amplification/deletion/mutation. Blue represents co-occurrent whereas red represents mutually exclusive. Fisher's exact test *: *P* < 0.05. (H and I) Kaplan-Meier survival curves for relapse-free survival (RFS) of patients with STK11/LKB1 mutations (H, *n* = 4) and *PTPRD* deletions (I, *n* = 8) versus wild type (Log-rank test). The false discovery rate (FDR) was used to correct the results of multiple comparisons. (J and K) Differentially mutated genes between (J) female and male, and (K) smoker and non-smoker. Fisher's exact test *: *P* < 0.05, **: *P* < 0.01, ***: *P* < 0.001, ****: *P* < 0.0001.

We collected 5676 surgical NSCLC specimens from 2007 to 2017 in Fudan University Shanghai Cancer Center, among which ∼2.1% (120/5676) were LUAS. Consistent with previous reports, LUAS patients showed relatively poor prognosis in contrast to either LUAD or LUSC patients (Fig. S1A). A total of 109 samples with high DNA and/or RNA quality were eventually used for further analyses: 93 LUAS with paired adjacent normal tissues were sequenced with WGS (tumor 60X; normal tissue 30X), and 93 tumors with 4 adjacent normal tissues were subjected to RNA-seq, among which 81 samples were analyzed with both WGS and RNA-seq. Moreover, we performed LCM on four samples containing well-separated adenomatous and squamous regions, and isolated pathology-defined components as well as paired adjacent normal tissues for WGS analyses (30X) (Fig. 1A and Table S1).

Among the 109 LUAS specimens, 71 (65.1%) were male and 38 (34.9%) were female. The proportion of non-smokers was 46.8%, slightly lower than two Asian LUAD cohorts (62.8% and 58%) [25,30]. The median age at surgery was 62 years (range: 32–84). The numbers of samples categorized by tumor stages were: I (55, 50.5%), II (19, 17.4%), III (34, 31.2%) and IV (1, 0.9%) (Table S1), which was significantly associated with relapse-free survival (RFS) (Fig. S1B).

### Somatic mutations in human LUAS

We first analyzed somatic single nucleotide variations (SNV) and small insertions or deletions using MuTect2 [45]. We found a median of 87 (range: 4–605) non-synonymous mutations per sample (Table S2) and a significant association between smoking status and high somatic mutations (*P* = 0.0001). Using MutSigCV, we identified 12 significantly mutated genes, containing known tumor suppressor genes such as *TP53, RB1, KEAP1, PTEN, STK11, P2RY8* and *CDKN2A*, and oncogenes such as *EGFR, ERBB2, KRAS, RUNX1T1* and *AKT1* [46,47] (Fig. 1B and Table S2). The most frequently mutated genes were *TP53* (59%) and *EGFR* (43%), similar to Asian LUAD [25,26]. The *EGFR* mutation rates of LUAS and LUAD were much higher than Asian LUSC (2%) [29]. The mutation burden was significantly lower (*P* = 0.0008) in *EGFR*-mutant LUAS but higher in *TP53*-mutant LUAS (*P* = 0.0055).

The mutation hotspots mainly occurred in oncogenes, including *EGFR, ERBB2, KRAS* and *AKT1* (Fig. 1C and D, Fig. S1C and D). Similar to LUAD, L858R mutation (50%, 20/40) and exon 19 deletion (25%, 10/40), which are known to be sensitive to *EGFR* tyrosine kinase inhibitors (TKI) [48], were the two most common *EGFR* genetic alterations in LUAS. Moreover, exon 20 insertion mutations were dominant in *ERBB2* mutations (57%, 4/7).

We further detected gene fusion events using RNA-seq data. We compiled a total of 66 in-frame gene fusions (Fig. S1E and Table S2), among which *ALK* fusions were most common (8%, 7/93). Six samples were with *EML4*-*ALK* fusions and one was with *KIF5B*-*ALK* fusion, and the expression of the *ALK* gene was significantly higher in these altered samples (Fig. S1F). We also found one sample with *KIF5B*-*RET* fusion (1%, 1/93). Moreover, we detected several previously categorized fusions in the integrated cancer fusion database (https://www.tumorfusions.org/), containing *ITGB6*-*RBMS1, TMEM123*-*MMP20* and *NF1*-*RNF135*. Despite its low alteration frequency, the TMEM123-MMP20 fusion, previously reported in the Cancer Genome Atlas (TCGA) data set, is associated with extremely high MMP20 gene expression (Fig. S1G), indicative of a potential function.

### Mutational signature analysis reveals three distinct patterns

Mutational spectrum analyses further revealed that the most common somatic substitutions in LUAS were C→T transition and C→A transversion (Fig. S1H), similar to LUAD [26,49]. Using the non-negative matrix factorization (NMF) algorithm, we identified three highly confident mutational signatures, named signature A, B and C respectively (Fig. 1E). Through the Pearson's correlation coefficient (PCC) analyses with the Catalogue of Somatic Mutations in Cancer (COSMIC) mutational signatures [47], we found that signature A was highly correlated with COSMIC signature 4 (cigarette smoking), which was frequently observed in LUAD and LUSC. In contrast, signature B correlated with COSMIC signature 1 (age of cancer diagnosis), and signature C correlated with COSMIC signature 13 (activity of the AID (activation-induced cytidine deaminase)/APOBEC (apolipoprotein B mRNA editing enzyme catalytic subunit) family) [47]. Unsupervised clustering divided human LUAS into three subgroups (Fig. S1I). Subgroup 1 was dominated by signature A (COSMIC signature 4), consistent with its high proportion of male smokers. Subgroup 2 was dominated by signature C, highly correlated with two APOBEC-associated signatures (COSMIC signatures 2 and 13). Subgroup 3 was dominated by signature B. Moreover, subgroup 1 showed frequent *KRAS* mutations and *SOX2*/*TP63* amplifications (Table S2). In contrast, subgroups 2 and 3 were enriched with *EGFR* mutations, which were rarely detectable in subgroup 1. Moreover, *ALK* fusions were mainly found in subgroup 3. In contrast to subgroups 2 and 3, subgroup 1 had very high tumor mutation burdens (TMBs) (*P* = 0.0044 and *P* < 0.0001, respectively).

### Somatic copy number variations in human LUAS

We next detected somatic copy number alterations (CNAs) and found a general CNA pattern in LUAS similar to TCGA LUAD and LUSC (Fig. 1F). Chromosomal regions containing *TERT, EGFR, MYC* and *ERBB2* were highly amplified, whereas the region containing *CDKN2A* was frequently deleted in LUAS, similar to the findings from TCGA LUAD and LUSC data sets. Interestingly, certain CNA segment alterations in LUAS were at an intermediate level between LUAD and LUSC, such as chromosome 3 (*SOX2* and *TP63*) and chromosome 22 (*MAPK1*). Notably, the *NXK2-1* allele showed the lowest rate of focal deletion in LUAS when compared with LUAD and LUSC. Significantly amplified peaks included those regions containing known oncogenes *EGFR* (7p11.2), *CCND1* (11q13.3) and *FGFR1* (8p11.23), as well as regions proximal to TERT (5p15.33), *MYC* (8q24.21) and *MDM2* (12q15). Peaks containing known tumor suppressor genes *CDKN2A* (9p21.3) and *PTEN* (10q23.31) were also frequently deleted (Fig. S1J, Table S3).

We further integratively analyzed concurrency and mutual exclusivity of top CNA and driver mutations (Fig. 1G). We found that *EGFR* mutations were mutually exclusive with *KEAP1* mutations and *ALK* fusions. *SOX2* amplifications were concurrent with *TP63* amplifications, *PIK3CA* amplifications and *CNKN2A* mutation, but mutually exclusive with *EGFR* mutations. *MDM2* amplification was concurrent with *EGFR* mutations and *CDKN2A* deletions, but mutually exclusive with *TP53* mutations.

Survival analyses indicated that patients harboring CNV deletion of *PTPRD* as well as somatic mutations in *STK11, CUL1, P2RY8* and *COL11A1* tended to have short RFS (Fig. 1H and I, Fig. S1K–M). *COL11A1* is reported to be increased in several cancers and high levels of *COL11A1* are often associated with poor survival, chemotherapy resistance and recurrence [50]. We found that four out of eight *COL11A1* mutations were located in the triple helix repeat region (Fig. S1N), indicative of potential oncogenic function, as previously reported [51]. *PTPRD* is a phosphatase involved in the JAK-STAT pathway and frequently inactivated in various types of cancers [52], and its deleterious alteration is associated with a lower survival rate and frequent metastasis [53–55]. Moreover, *TP53* mutations were enriched in males and *KMT2D* mutations were associated with gender and smoking status (Fig. 1J and K). In contrast, *EGFR* mutations were significantly enriched in females and non-smokers in LUAS, similar to those observed in LUAD [28,49].

### Monoclonal origin and potential evolution path of LUAS

We further compared the mutation spectrum of LUAS with Asian LUAD and LUSC [29,30] (Fig. S2A and B). Our data revealed a high correlation between LUAS and LUAD, whereas the correlation between LUAS and LUSC was relatively low. Specifically, the high rate of oncogenic driver mutations of *EGFR* in LUAS was comparable to LUAD but not LUSC. This indicated that LUAS more resembled LUAD.

**Figure 2.**
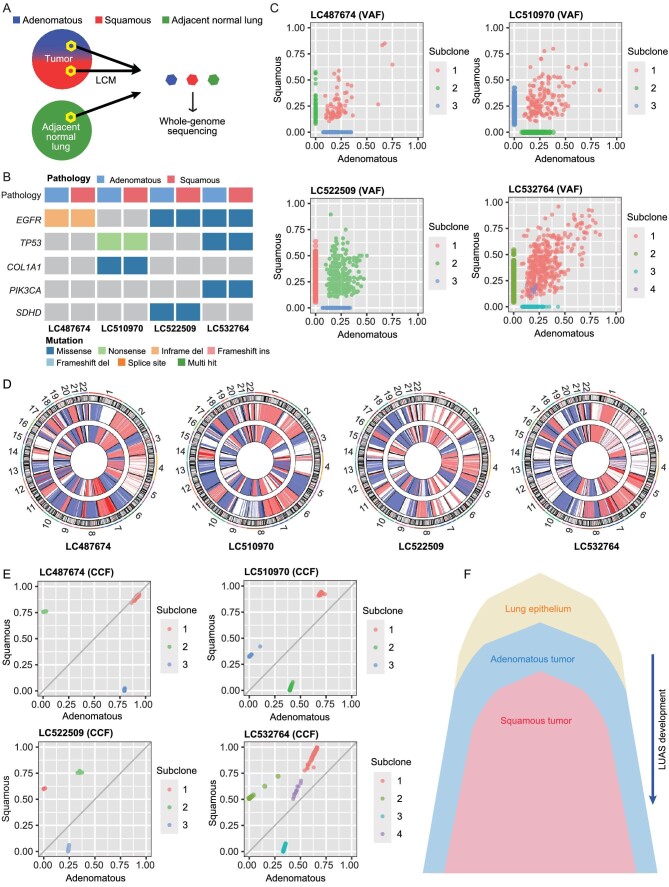
The potential evolutional path of Chinese LUAS. (A) Scheme of laser-capture microdissection of four LUAS for WGS analyses. (B) Somatic mutation plot of top cancer-related genes in paired adenomatous and squamous components of Chinese LUAS specimens. (C) Variant allele frequency (VAF) plot of somatic mutations between paired adenomatous and squamous components. (D) Circos plot of somatic CNVs between paired adenomatous and squamous components using CNV scores generated with GISTIC2.0. (E) Cancer cell fraction (CCF) plot of somatic mutations between paired adenomatous and squamous components. (F) Linage transition model for LUAS development.

To investigate the potential evolution path of LUAS, we further analyzed four samples containing well-separated adenomatous and squamous components, and isolated them via LCM for subsequent WGS analyses together with paired adjacent normal tissues (Fig. 2A). We found that most top cancer-related mutations: *EGFR* and *PIK3CA*, were shared across matched adenomatous and squamous pathologies in all four samples (Fig. 2B, Fig. S2C and Table S4). The comparison of the variant allele frequency of somatic mutations at the whole-genome scale, as well as somatic CNV patterns, also showed a high concordance between paired adenomatous and squamous components (Fig. 2C and D). These findings indicate that the adenomatous and squamous pathologies in LUAS are likely derived from a monoclonal origin (Fig. S2D). The high number of private mutations in adenomatous or squamous components might be ascribed to early separation of these two different pathologies during LUAS microevolution.

To study the potential lineage transition, we plotted the cancer cell fractions (CCFs) based on the mutation information to deduce the evolutionary route between adenomatous and squamous pathologies (Fig. 2E). Most of these samples owned a cluster of mutations, which tended to be clonal in squamous pathology, and subclonal in adenomatous pathology, indicating that adenomatous subclones might appear first and subsequently transdifferentiate into squamous lesions (Fig. 2F) [56]. Moreover, this lineage-transition working model is in agreement with recent studies [10,13].

### Somatically altered oncogenic signaling pathways

**Figure 3.**
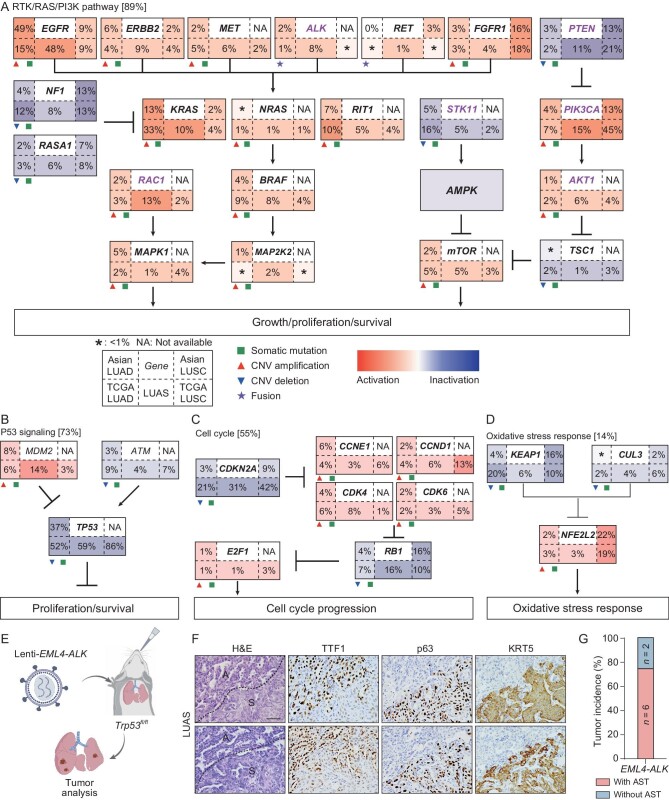
Somatically altered oncogenic signaling pathways in Chinese LUAS. (A–D) Somatic mutations, copy number alterations and gene fusions involved in (A) RTK/RAS/PI3K pathway, (B) P53 signaling, (C) cell cycle and (D) oxidative stress response. Each gene box included five indexes representing the alteration frequencies of activation and inactivation in (top-left) Asian LUAD, (bottom-left) TCGA-LUAD, (bottom-middle) Chinese LUAS, (top-right) Asian LUSC and (bottom-right) TCGA-LUSC. Asterisks (*) represent altered frequency at <1%. (E) Schematic diagram of the *EML4-ALK* mouse model study. (F) Representative H&E and TTF1 (gene name *NKX2-1*), p63 and KRT5 immunostaining photos of *EML4-ALK* fusion-driven mouse LUAS. Scale bar: 50 μm. (G) Quantification of AST incidence in the *EML4-ALK* mouse model (*n* = 8).

We next analyzed oncogenic signaling pathways through the integration of somatic mutations, gene fusions and CNAs (Fig. 3A–D and Fig. S3A–D) [57]. Similar to LUAD and LUSC, the most frequently altered pathway in LUAS was the RTK/RAS/PI3K pathway (89%) (Fig. 3A). Moreover, P53 signaling (73%) and cell cycle (55%) were also frequently altered (Fig. 3B and C). The oxidative stress response pathway (*NFE2L2, KEAP1, CUL3*), important for both LUAD and LUSC [27,28], was altered in 14% of LUAS (Fig. 3D).

We further compared LUAS with TCGA LUAD (PanCancer Atlas, *n* = 507) and Asian LUAD (*n* = 302) cohorts, as well as with TCGA LUSC (PanCancer Atlas, *n* = 469) and Asian LUSC (*n* = 104) cohorts to identify potential oncogenic drivers involved in LUAS development [29,30]. Among those altered genes in the RTK/RAS/PI3K pathway (Fig. 3A), *RAC1, ALK* and *AKT1* alterations were significantly enriched in LUAS (compared to Asian LUAD, *P* = 0.0004/0.02/0.008). Among which, AKT1 has been recently reported to regulate the AST [11]. The *STK11/LKB1* mutation rate was relatively low in LUAS. This might be due to the ethnic difference since Asian LUAD also shows a lower mutation rate in contrast to Caucasian LUAD [28,30]. Asian LUAD tends to have high rates of EGFR mutations (48%), which is known to be mutually exclusive with STK11 mutations [42–44]. Moreover, mutations of many genes in LUAS such as *PIK3CA, RASA1* and *PTEN* showed intermediate rates between LUAD and LUSC. In the P53 signaling pathway, *MDM2* mutations were specifically enriched in LUAS (Fig. 3B). In the cell cycle pathway, *CDKN2A, CCND1* and *CDK6* showed intermediate mutation rates whereas *RB1* had higher mutation rates in LUAS (Fig. 3C).

Consistent with previous studies [11,58], AKT, MYC and STK11 are implicated as important regulators of squamous transdifferentiation. Interestingly, no study has reported the role of ALK fusion in AST, despite the fact that ALK fusion LUAD shows diverse pathological patterns [59–61]. Some ALK fusion LUADs display expression of p63 (gene name *TP63*), the marker of LUSC [60,62]. To test the potential role of ALK fusion in LUAS development, we treated the *Trp53^fl/fl^*mice with lentivirus carrying the EML4-ALK fusion (a drug-resistant mutant L1196M) (Fig. 3E). Knockout of Trp53 alone is known to be insufficient for driving lung tumorigenesis. We found that the majority of lung tumors displayed adenocarcinoma pathology and were positive for TTF1 staining (Fig. 3F). Moreover, we also detected some tumors with mixed adenomatous and squamous pathologies, which were diagnosed as LUAS (Fig. 3F). This implies a strong link between EML4-ALK and LUAS development. The penetrance of squamous transdifferentiation was as high as 75% (Fig. 3G). These data identify ALK fusion as a novel oncogene in driving LUAS development.

### Identification of the inflammatory subtype as the intermediate state of AST

**Figure 4.**
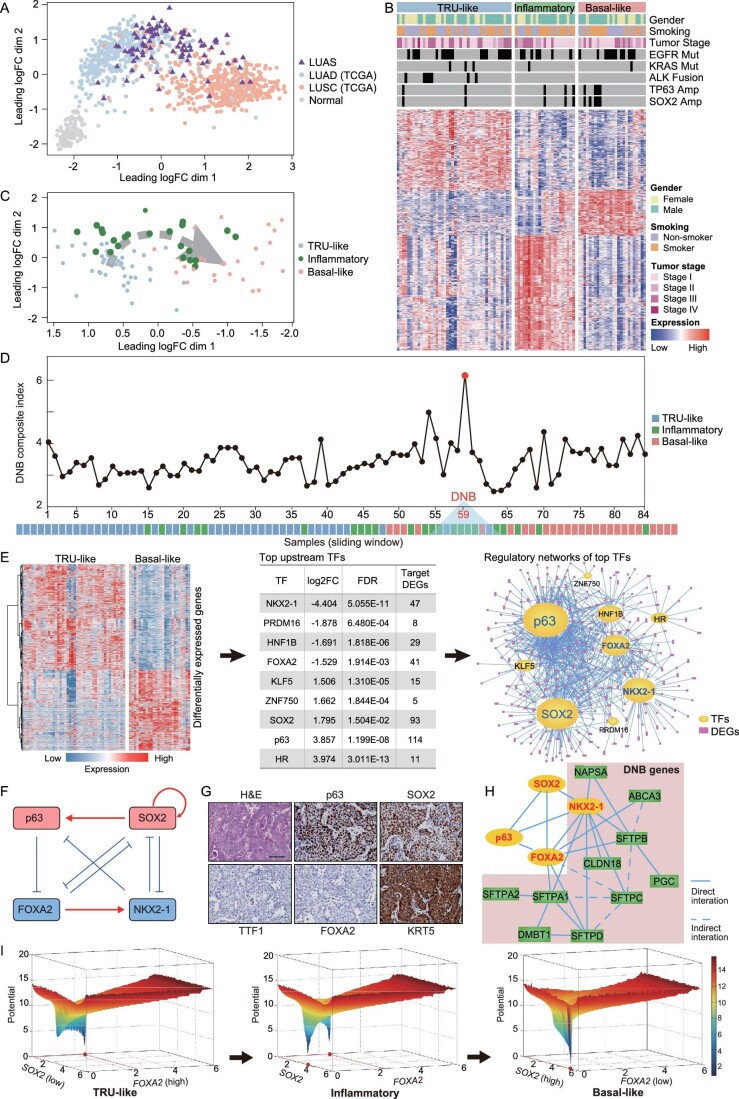
Identification of the inflammatory type as the intermediate stage during LUAS development. (A) Multidimensional scaling (MDS) plot of LUAS RNA-seq data together with TCGA LUAD and LUSC data. (B) Unsupervised clustering classified LUAS into three different classes. Clinical features, genetic features and expressions of signature genes in each class were indicated. (C) MDS plot of three classes of LUAS. (D) Composite index of window-sliding dynamic network biomarker (DNB) analyses performed on ranked samples with the increase of LUSC GVSA score. The tipping point, which was represented by 10 samples (from 59 to 68) in one sliding window, was highlighted in red. (E) Heatmap of differentially expressed genes (DEGs) between TRU-like subtype and basal-like subtype (left). Top upstream transcription factors (TFs) inferred by IPA through the DEG analyses (middle). Regulatory network of top TFs together with directly linked DEGs (right). Hub TFs were highlighted in bold. (F) The core regulatory network is composed of four lineage-counteracting TFs: two adenomatous-lineage-specific TFs *NKX2-1* and *FOXA2* counteract with two squamous-lineage-specific TFs *TP63* and *SOX2*. (G) Representative H&E and immunostaining photos of squamous lung tumors derived from *Kras^G12D/+^; Rosa26^LSL-Cas9^* mice post 28 weeks of lenti-sg*Tomato* (sg*Tom*) (*n* = 5) or lenti-sg*Nkx2-1*-sg*Foxa2* (sgN+F) (*n* = 5) viral infection through nasal inhalation. Scale bar: 50 μm. (H) Transcriptional regulatory network of core TFs in context with DNB genes. (I) Potential energy landscapes of three stages/classes/subtypes of LUAS. The lowest point had the highest probability of cell state (type) whereas the highest point had the lowest probability of cell state. Potential (Equation (S3)) was defined as -ln(PSS) or -ln(cell state probability), which was estimated by stochastic simulation based on the mathematical model (Equation (S2)) of four core TFs. See supplementary methods for details.

We next performed multidimensional scaling (MDS) analyses of the LUAS RNA-seq data together with TCGA LUAD and LUSC data (Fig. 4A and Table S5). We found that most LUAS were scattered in between LUAD and LUSC samples. Through unsupervised clustering, we identified three mRNA-based classes [63] (Fig. 4B and Table S5). Interestingly, LUAD markers *NKX2-1* and *NAPSA*, were highly expressed in Class 1 whereas LUSC markers *TP63, KRT5* and *DSG3*, were highly expressed in Class 3 [64–66]. Further analyses showed that Class 1 shared molecular signatures with the TRU subtype in TCGA LUAD in comparison with the proximal inflammatory and proximal proliferative subtypes (Fig. S4B) [28]. Moreover, *ALK* fusion was significantly enriched in Class 1, similar to the TRU subtype (Fig. S4C) [28]. Class 3 was similar to the basal subtype of TCGA LUSC (Fig. S4B) [27]. Class 2 was quite unique. It was similar to the TRU-I (TRU-inflammatory) subtype in Asian LUAD and significantly enriched with immune-related pathways such as chemokine signaling, T cell receptor signaling and B cell receptor signaling [30] (Fig. S4A and B and Table S5). We thereafter referred to Class 1 as the TRU-like subtype, Class 2 as the inflammatory subtype and Class 3 as the basal-like subtype. Our MDS analyses of these three classes also suggested that the inflammatory subtype might be at the intermediate stage between the TRU-like subtype and the basal-like subtype (Fig. 4C). This is further supported by the analyses of adenomatous and squamous markers across various LUAS subtypes (Fig. S4D and E) [65].

To further evaluate the transcriptomic dynamics of LUAS, we took advantage of the dynamic network biomarker (DNB) theory, previously used for identifying the tipping point in various biological transition processes [67–69]. Through computing top differentially expressed genes (DEGs) between TCGA LUAD and LUSC, we established the lineage-specific gene set variation analysis (GSVA) scores and aligned the 93 LUAS samples with the GSVA scores (Fig. S4F and G and Table S5). As expected, the expression of known LUAD markers decreased gradually whereas the expression of known LUSC markers increased in these ranked LUAS samples (Fig. S4H). The inflammatory subtype fell in between the TRU-like and the basal-like subtypes, further confirming its potential role as the intermediate stage. Using the sliding-window DNB analyses in these ordered samples [68] (details in supplementary methods), we identified the tipping point composed of ∼10 samples (the 59th window), among which 7 belonged to the inflammatory subtype (Fig. 4D, total 31 DNB genes shown in Table S5). These data collectively identify the inflammatory subtype as the intermediate stage during LUAS development.

We further performed the deconvolution of LUAS gene expression using LUAD and LUSC signature genes to evaluate the adenocarcinoma/squamous ratios (Fig. S4I–K). We performed Pearson correlation analysis between the GSVA scores of seven immune cell types and the adenocarcinoma/squamous ratio, and found no significant correlation between tumor microenvironment (TME) components and the adenocarcinoma/squamous ratios (Fig. S4L). Instead, we observed that the mutation of KMT2D and CNV amplification of SOX2 were significantly associated with high adenocarcinoma/squamous ratios (Fig. S4M).

### Dynamical interaction among the core transcription factors in LUAS

We next performed comparative gene expression analyses between the TRU-like subtype and basal-like subtype to reveal those potential molecular signatures involved in lineage transition (Fig. 4E). Using ingenuity pathway analysis (IPA) [70] based on the DEGs, we identified the nine most significantly deregulated upstream transcription factors (TFs) (Fig. 4E and Table S6). Through detailed gene transcriptional network analyses of these TFs and DEGs, we identified four lineage-specific TFs containing LUAD markers NKX2-1 and FOXA2, and LUSC markers SOX2 and p63 as the hub TFs (Fig. 4E) [65]. The IPA analyses showed that this four-TF regulatory network formed a reciprocal feedback and feedforward loop structure [71,72], except for the regulatory relationship between FOXA2 and SOX2. Using *Kras^G12D/+^* mouse embryonic fibroblasts (MEFs) and *Kras^G12D^; Trp53^−^^/^^−^* (KP) mouse lung cancer cell lines [73], we demonstrated that *Foxa2* knockout significantly up-regulated SOX2 (Fig. S5A–C). With the integration of these data, we established the ‘core’ TF network in LUAS development (Fig. 4F), in which two adenomatous-lineage TFs counteracted two squamous-lineage TFs. To test if simultaneous deletion of two adenomatous-lineage TFs drives LUAS initiation, we used the clustered regularly interspaced short palindromic repeats (CRISPR)/Cas9 system to knockout both *Nkx2-1* and *Foxa2* in a *Kras^G12D^*-driven LUAD mouse model. Consistent with a previous study [74], we detected AST in two out of five mice (Figs 4G and S5D) and these transitioned tumors were more malignant and fast-growing (Fig. S5E and F). A previous study also supports the essential role of SOX2 in driving squamous transdifferentiation [75]. These data collectively support the essential role of the four-TF counteracting network during LUAS development.

Intriguingly, NKX2-1 itself belongs to the scope of DNB-related genes, and among four core TFs, NKX2-1 and FOXA2 showed a strong connection with DNB genes (Fig. 4H). We further deconvoluted the dynamic progression processes during LUAS development via measuring the relative weights of three components: the adenomatous-lineage TFs (NKX2-1 and FOXA2), the squamous-lineage TFs (SOX2 and p63) and the inflammatory or immune signature genes (Fig. S5G). We found that typical lineage transition during LUAS development potentially included three steps: initially the LUAD signature decreased, and then the inflammatory signature increased drastically at the tipping point, and eventually the LUSC signature increased and dominated (Fig. S5H).

Based on the regulatory relationship of these four core TFs (Fig. 4F), we further constructed a mathematical model for studying cell population dynamics. Our model qualitatively confirmed not only biological experiments but also bi-stable dynamics, i.e. adenomatous state and squamous state, formed by non-linear counteractions of two adenomatous-lineage TFs and two squamous-lineage TFs (details in supplementary methods). The potential energy landscape analysis by changing parameter ${\alpha }_S$ of the mathematical model (bifurcation analysis) indicated that cancer cells were initially maintained at the adenomatous state, gradually progressed into an inflammatory state which is an intermediate transition stage mixed with both adenomatous and squamous states and eventually reached the squamous state (Fig. 4I, Fig. S5I and Table S6).

### CXCL3/5-mediated neutrophil infiltration promotes AST

**Figure 5.**
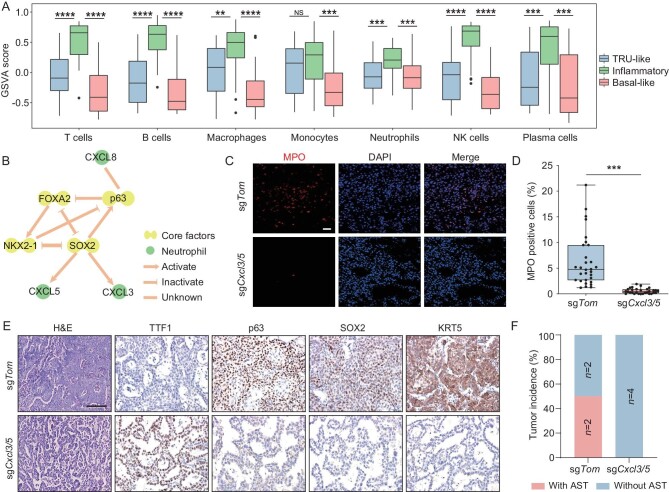
Neutrophil recruited by CXCL3 and CXCL5 regulates the dynamical transition of LUAS. (A) Comparison of GSVA scores for seven immune cell types in different LUAS classes. Student's t-test, *P < 0.05, **P < 0.01, ***P < 0.001, ****P < 0.0001. (B) Transcriptional regulatory network of four core TFs, DNB genes and chemokine genes. (C) Immunostaining for neutrophil marker myeloperoxidase (MPO) in *Kras^G12D/+^; Lkb1^fl/fl^; Rosa26^LSL-Cas9^* mice post 16 weeks of lenti-sg*Tomato* (sg*Tom)* (*n* = 4) and lenti-sg*Cxcl3*+*Cxcl5* (sg*Cxcl3/5*) (*n* = 4) viral infection through nasal inhalation. Scale bar: 100 μm. (D) Quantification of the percentage of MPO positive cells in *Kras^G12D/+^; Lkb1^fl/fl^; Rosa26^LSL-Cas9^* mice treated with sg*Tom* or sg*Cxcl3/5*. (E) Representative H&E and immunostaining photos from lung tumors in *Kras^G12D/+^; Lkb1^fl/fl^; Rosa26^LSL-Cas9^* mice treated with sg*Tom* and sg*Cxcl3/5*. Scale bar: 50 μm. (F) Quantification of AST incidence in *Kras^G12D/+^; Lkb1^fl/fl^; Rosa26^LSL-Cas9^* mice treated with sg*Tom* or sg*Cxcl3/5*. ‘without AST’ means only lung adenocarcinoma detectable.

We further found that the GSVA scores of seven immune cell types, including neutrophils, T cells and B cells, were significantly higher in the inflammatory subtype (Fig. 5A) [76,77]. Consistently, the GSVA scores for chemokines and their receptors involved in inflammatory cell infiltration were also the highest in the inflammatory subtype [78] (Fig. S6A). We further confirmed that SOX2, one of the core TFs, positively regulated the expression of CXCL3 and CXCL5, two chemokines known to recruit neutrophils into the TME (Fig. 5B and Fig. S6B–E) [72]. Concurrent knockout of *Cxcl3* and *Cxcl5* in the *Kras^G12D/+^; Lkb1^fl/fl^; Rosa26^LSL-Cas9^* mouse model significantly diminished neutrophil infiltration (Fig. 5C and D). More importantly, *Cxcl3/5* knockout resulted in the suppression of AST (Fig. 5E and F). These data highlight the important role of chemokine-mediated neutrophil infiltration in mediating AST.

### LSD1 is a potential therapeutic target of LUAS

The RNA-seq data enable us to explore the evolution path during LUAS development. In contrast to gene expression profiling, which can be largely affected by sampling variation, the genomic changes are relatively stable. To explore the relationship between genetic alterations and clinical relevance, we have integrated the somatic mutations and copy number variations and classified LUAS into three major classes: C1, C2 and C3 (Fig. S7A and B). We found that C1 featured cell differentiation and abnormal development signaling, e.g. the amplification of multiple HOX family members: HOXA3/9/11/13, which were important for embryogenesis [79]. Specifically, ALK fusion, MDM2 and RAC1 amplification were enriched in C1. About 83.3% (5/6) of ALK fusions were found in C1. C2 was enriched with a basal-like signature, e.g. frequent amplification of squamous-related TFs SOX2 and TP63. The EGFR mutation rate was lower in C2 compared to C1 and C3. In contrast, C3 featured receptor tyrosine kinase (RTK) signaling and an epigenetic program, e.g. ERBB2, AKT1, ARID1B and SETD2 mutations. We further compared the relationship among RNA, DNA and mutational signature-based classifications. According to the result, these three transcriptome subgroups did not correlate significantly with DNA subgroups. In addition, DNA subgroups and mutational signature-based subgroups had a certain relationship: DNA-C2 was more consistent with MS-C1, DNA-C3 was more consistent with MS-C3, and DNA-C1 could be split into MS-C2 and MS-C3 (Fig. S7C).

**Figure 6.**
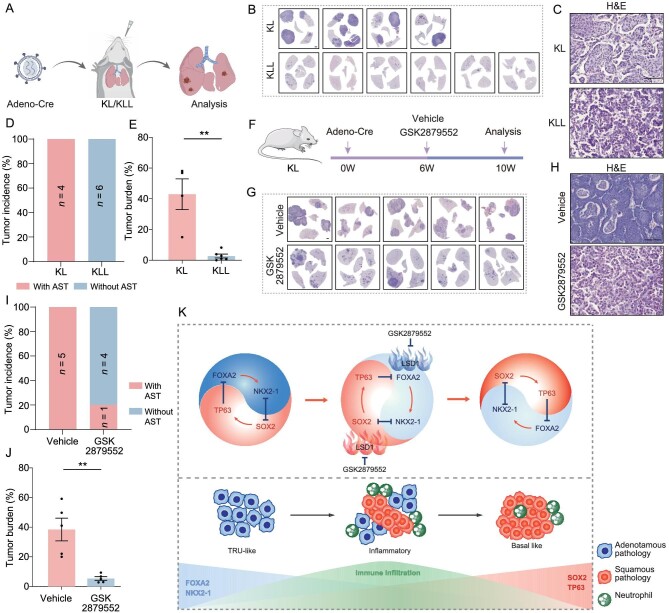
Identification of LSD1 as a potential therapeutic target in STK11/LKB1-mutated LUAS. (A) Schematic diagram of the *Kras^G12D/+^; Lkb1^fl/fl^* (KL) and *Kras^G12D/+^; Lkb1^fl/fl^; Lsd1^fl/fl^* (KLL) mouse model. (B) Images of H&E staining for individual lungs from KL (*n* = 4) and KLL (*n* = 6) mice. (C) Representative H&E staining of KL and KLL tumors. Scale bar: 50 μm. (D and E) Quantification of (D) AST incidence and (E) tumor burden in KL and KLL mice post 10 weeks of Adeno-Cre viral infection through nasal inhalation. Data are shown as mean ± SEM. **P < 0.01. (F) Schematic diagram of the treatment strategy in the KL model. (G) Images of H&E staining for individual lungs of KL mice treated with vehicle or GSK2879552. (H) Representative H&E staining in KL mice treated with vehicle (*n* = 5) or GSK2879552 (*n* = 5). Scale bar: 50 μm. (I and J) Quantification of (I) AST incidence and (J) tumor burden of KL mice treated with vehicle (*n* = 5) or GSK2879552 (*n* = 5). Data are shown as mean ± SEM. **P < 0.01. (K) Working model for dynamic lineage transition during LUAS development.

STK11 is found to be specifically enriched in C3. In contrast to C1 and C2, C3 was significantly associated with the worst prognosis (Fig. S7D). To identify the therapeutic vulnerability of this specific subset of *STK11*-mutated LUAS, we focused the epigenetic regulators based on its potential link with AST [11]. Our analysis of tumor RNA-seq data from the mouse AST model (*Kras^G12D/+^; Lkb1^fl/fl^*, KL) showed that multiple epigenetic regulators including *LSD1* (lysine-specific histone demethylase 1, also known as *KDM1A*) were up-regulated in squamous lesions compared to adenomatous lesions (Fig. S7E). Consistently, *LSD1* expression was up-regulated in human LUSC vs. LUAD (TCGA data sets) (Fig. S7F) and it is significantly correlated with the GSVA scores of our core-TF network (Fig. S7G and H). LSD1 is known to regulate lineage-specific genes SOX2 and FOXA2 [80]. We found that *Lsd1* deletion in the KL model completely blocked the AST process (Fig. 6A and B). The *Kras^G12D/+^; Lkb1^fl/fl^; Lsd1^fl/fl^* (KLL) model exclusively exhibited LUAD pathology (Fig. 6C). Moreover, the AST incidence and tumor burden were also dramatically decreased (Fig. 6D and E). These data demonstrate that *Lsd1* knockout blocks the AST process.

GSK2879552, an irreversible inhibitor of LSD1, is reported to effectively suppress small cell lung cancer (SCLC) [81]. To assess whether LSD1 could be a potential therapeutic target in LUAS, we treated the KL model with GSK2879552 (Fig. 6F). Similar to *Lsd1* knockout, GSK2879552 treatment obviously inhibited KL tumor progression and neutrophil infiltration (Fig. 6G and Fig. S7I). Most mice showed LUAD pathology, and AST incidence and tumor burden were dramatically decreased (Fig. 6H–J). These data thus identify the therapeutic vulnerability of STK11-mutated LUAS, which might have therapeutic implications for clinical treatment.

We here uncover the potential mechanisms of lineage transition in human LUAS involving dynamic changes of the core four-TF network and immune infiltration (Fig. 6K). NKX2-1 and FOXA2 maintain the adenomatous lineage whereas SOX2 and p63 maintain the squamous lineage. SOX2 expression is important for CXCL3 and CXCL5 production and neutrophil recruitment. Moreover, we also identify a novel therapeutic strategy involving LSD1, which strongly correlates with the core-TF network.

## DISCUSSION

Human LUAS is notorious for its high malignancy and strong cancer plasticity despite its small percentage in lung cancer. Systematic analyses of human LUAS genomics and transcriptomics is urgently needed to uncover the underlying mechanism and identify therapeutic vulnerability with the hope of improving clinical treatments. Through a 10-year effort, we have obtained 109 human LUAS from over 5000 surgical NSCLC samples and performed systematic characterization of its genomic alterations, transcriptomic dynamics and evolutionary features, thus providing an up-to-date comprehensive genomic and transcriptomic landscape of human LUAS. Accumulating clinical case reports as well as mouse-model studies has recently supported the lineage transition hypothesis for LUAS development [39,82]. Consistent with these previous studies [10,11], we find that LUAS is very likely derived from a monoclonal origin and developed from adeno-to-squamous transdifferentiation.

LUAS is a mixture of two pathological components, which makes it very difficult to dissect single pathology. This hinders the LUAS classification of various subtypes simply via gene expression profiling, which could be potentially affected by sampling variation. Nonetheless, we can take advantage of gene expression profiling analyses to decipher biological dynamics during the AST process. Through the consensus clustering of gene expression profiling, we have divided LUAS into three distinct subtypes: the TRU-like, the inflammatory, and the basal-like subtypes. We further identify the inflammatory subtype that features enhanced immune infiltration as the intermediate stage for AST. Lineage-defining TFs are known to control cancer cell identity [72,83,84]. Interestingly, we find that the four lineage-defining TFs, i.e. *NKX2-1, FOXA2, SOX2* and *TP63*, form a counteracting regulatory network to control the development of LUAS. *NKX2-1* and *FOXA2* maintain the adenomatous lineage. On the other hand, *SOX2* and *TP63* maintain the squamous lineage [74,85–87]. These four TFs are counteracting through forming multiple feedforward and feedback loops. Our work, together with previous studies, supports the theory that the combined modulation with at least two factors might be important for disrupting homeostasis and driving squamous transdifferentiation, e.g. concurrent knockout of both adenomatous-lineage TFs *Nkx2-1* and *Foxa2* or, overexpression of *Sox2* plus *Nkx2-1* knockout, drives AST [74,75]. Inflammatory factors might also contribute to LUAS development. A recent report has demonstrated that neutrophil promotes the squamous transdifferentiation in a mouse model [88]. We and others show that *SOX2* promotes expression of *CXCL3* and *CXCL5*, the chemokines for neutrophil recruitment [72]. When both *Cxcl3* and *Cxcl5* were simultaneously deleted in a KL mouse model, the AST process was significantly suppressed. Our data establish the evolutionary path of AST and illustrate the essential roles of the core four-TF network and immune infiltration in LUAS development, which is further supported by the bifurcation analysis and energy landscape analysis in our mathematical model.

Our study further identifies the oncogenic drivers involved in the AST process including STK11/LKB1, AKT1 and MYC [39,58]. The low rate of STK11 mutations in LUAS is likely due to its mutually exclusivity with EGFR mutations as well as ethnic difference [42–44]. AKT1 and MYC, recently reported as oncogenic drivers in LUAS [11], are also observed enriched in Chinese LUAS. Besides these known drivers, we identify RAC1 and ALK as potential candidates for LUAS oncogenic drivers. Increased *RAC1* activity has been associated with TKI resistance and lung cancer metastasis [89,90]. It will be interesting to see if future efforts test whether RAC1 indeed regulates AST.

We further find that ALK fusion is enriched in ∼8% (7/93) of LUAS vs. 1%–2% in two large LUAD cohorts (TCGA PanCancer, *n* = 566; East Asia (EAS), *n* = 302). Previous studies show that AST is observed in ALK-rearranged patients after the acquisition of TKI resistance [91–98]. Our mouse model study shows that ALK overexpression is able to drive lung adenosquamous carcinoma development. Our ongoing work also demonstrates that a club cell, but not type II pneumocyte, is the major cell of origin of lung adenosquamous carcinoma (data not shown). This might explain the previously unappreciated role of ALK fusion in adenosquamous carcinoma development, since most mouse models were based on the origin of a type II pneumocyte [99,100]. A previous study generated the CRISPR/Cas9-mediated EML4-ALK rearrangement in mice without notable adenosquamous carcinoma observation [101]. We reason that this could be due to several reasons: (i) an unusually high amount of adenovirus (1.5 × 10^8^ p.f.u.) was used for intratracheal instillation, since the incidence of rearrangement event is presumably low; (ii) the potential toxicity of high Cas9 expression as previously reported [102,103] might affect the AST occurrence; (iii) due to a high amount of tumor burden, most mice have to be sacrificed around 12 weeks, which might not be long enough for AST occurrence. It will be interesting if future efforts clarify these factors and test whether AST exists in this model. The major downstream signaling pathways of EML4-ALK fusion include the mitogen-activated protein kinase (MAPK), the phosphoinositide-3-kinase (PI3K) and the signal transducer and activator of transcription 3 (STAT3) pathways [104]. Interestingly, recent studies demonstrate that the JAK-STAT signaling is a crucial executor in regulating lineage plasticity in prostate cancer [105,106]. Moreover, another study demonstrates that PI3K/AKT and MYC activation induce squamous features and the expression of LUSC markers in EGFRmutant LUAD preclinical models [11]. Our work here uncovers the potential link between ALK fusion and LUAS development. It will be interesting, in the future, to explore the potential molecular mechanisms downstream of EML-ALK that are involved in the AST process.

In contrast to RNA-seq data, the genetic alterations are more stable and not easily affected by the sampling variation, which makes the clustering of LUAS feasible. Through integrative subtyping based on the somatic mutation and copy number variation data, we have classified LUAS into three classes with distinct features. Among them, ALK fusion mainly occurs in Class 1 whereas TP63/SOX2 amplification is enriched in Class2. Importantly, Class 3 is enriched with STK11 mutation and is associated with the worst prognosis. Previous studies have demonstrated that STK11-mutant lung tumors are very malignant, highly plastic and resistant to various therapies [39,58,88,107–109]. Detailed analyses of the malignant Class 3 identify *LSD1* as an epigenetic modifier highly correlated with the core four-TF network. As previously reported, lineage plasticity is associated with epigenetic alterations such as EZH2 [11]. LSD1, a histone demethylase, is known to regulate lineage-specific genes SOX2 and FOXA2 by selectively modulating the methylation states of histone H3 at lysines 4 (H3K4) and 9 (H3K9) [80]. Of note, the proteomic analysis of human LUSC provides a rationale for exploring chromatin modifiers such as LSD1 and EZH2 to target SOX2-overexpressing LUSC [31]. We find that *Lsd1* knockout almost completely blocks the AST in the KL mouse model. Importantly, LSD1 inhibitor treatment also drastically suppresses tumor progression in mice. Multiple LSD1 inhibitors have been developed in preclinical and clinical trials to treat hematological malignancy and solid cancer [81,110,111]. Thus, it remains practical to test our strategy in the future treatment of STK11-deficient lung cancer in the clinic.

## METHODS

### Clinical sample collection and genomic and transcriptional sequencing

Primary tumor specimens and paired adjacent normal tissues were obtained from LUAS patients who underwent surgical resection in the Department of Thoracic Surgery, Fudan University Shanghai Cancer Center. Tumors and adjacent normal tissues were snap frozen and stored in liquid nitrogen upon resection until later use. The tumor specimens were reviewed by two pathologists independently to determine the histological subtype and Tumor, Lymph Node, Metastasis (TNM) stage. All patients who participated in this study provided written informed consent. All the enrolled specimens were treatment-naïve and no therapy before surgery was applied. A total of 5676 surgical NSCLC tumors were collected from 2007 to 2017 containing 120 LUAS, 3295 LUAD, 893 LUSC and 1488 other subtypes. All the LUAS samples were defined by pathologists, with the presence of at least 10% of adenomatous pathology and squamous pathology in single tumors. Eleven samples were excluded due to poor genomic DNA and/or RNA quality, and 109 samples were eventually used for genomic and transcriptomic sequencing. Among these 109 samples, 93 tumors with paired adjacent normal tissues were sequenced with WGS (tumor 60X; normal tissue 30X). LCM was performed on four samples with separate adenomatous and squamous components to isolate genomic DNA from different pathologies, and the samples were also subjected to WGS analyses together with paired adjacent normal tissues (30X). A set of 93 tumors with 4 normal lungs were RNA sequenced. In total, 81 samples were analyzed with both WGS and RNA-seq.

### Mouse study

*Kras^G12D/+^, Trp53^fl/f^^l^* and *Lkb1^fl/fl^* mice were originally generously provided by Dr. Tyler Jacks [112] and Ronald Depinho [113]. An *Lsd1^fl/fl^* mouse was generously provided by M. Rosenfeld [114]. A *Rosa26^LSL-Cas9^* mouse was purchased from Shanghai Model Organisms Center. All mice were housed in a specific pathogen-free environment at the CAS Center for Excellence in Molecular Cell Science, Chinese Academy of Sciences, and treated in strict accordance with protocols approved by the Institutional Animal Care and Use Committee of the CAS Center for Excellence in Molecular Cell Science, Chinese Academy of Sciences. The adenovirus and lentivirus were delivered to mice via nasal inhalation and lung tumor pathologies were analyzed as previously described [73]. To validate the capability of *EML4-ALK* to drive LUAS, the *Trp53^fl/fl^* mice infected with Lenti-*EML4-ALK^L1196M^* (1 * 10^∧^6 p.f.u) were analyzed at 18 weeks post-treatment. To test if simultaneous deletion of *Nkx2-1* and *Foxa2* drives squamous transition, *Kras^G12D/+^; Rosa26^LSL-Cas9^* mice were treated with the lentivirus of sg*Tomato* (sg*Tom*) or sg*Nkx2-1*-sg*Foxa2* (sgN+F) (2 * 10^∧^6 p.f.u) and analyzed at 28 weeks post-treatment. To knockout both *Cxcl3* and *Cxcl5, Kras^G12D/+^; Lkb1^fl/fl^; Rosa26^LSL-Cas9^* mice were treated with the lentivirus of sg*Tom* or sg*Cxcl3*+*Cxcl5* (sg*Cxcl3/5*) (2 * 10^∧^6 p.f.u) and analyzed at 16 weeks post-treatment. The sgRNAs of *Tomato* and *Nkx2-1* were chosen as previously reported [72,115]. The sgRNAs of *Foxa2, Cxcl3* and *Cxcl5* were designed using optimized CRISPR Design (http://chopchop.cbu.uib.no/). The knockout efficiency of sg*Foxa2* was analyzed as previously described [73]. The sequences of all sgRNAs and the primers for knockout efficiency detection are included in Table S6. *Kras^G12D/+^; Lkb1^fl/fl^; Lsd1^fl/fl^*mice were treated with Adeno-Cre (2 * 10^∧^6 p.f.u) and analyzed at 10 weeks post-treatment. For pharmacological treatment in *Kras^G12D/+^; Lkb1^fl/fl^* mice, either a vehicle or GSK2879552 (1.5 mg/kg) (HY-18632, MedChemExpress) was given to mice at 7–9 weeks post-Adeno-Cre treatment via intraperitoneal injection, daily, for four weeks.

### Statistical analyses

Comparisons of mutation burdens were performed with the Wilcoxon rank-sum test and *P* < 0.05 was considered significant. Fisher's exact test was used to detect concurrent and mutually exclusive events as well as significantly enriched genomic features in different subgroups. Differentially expressed genes were calculated with Student's t-test and adjusted using the Benjamini-Hochberg (BH) method. All experimental data were analyzed via Student's t-test and *P* < 0.05 was considered to be significant.

### DATA AVAILABILITY

Raw WGS data and RNA-seq data are available via the National Omics Data Encyclopedia (NODE, OEP001032).

## Supplementary Material

nwad028_Supplemental_Files
