# Supplementary material for: Counteracting lineage-specific transcription factor network finely tunes lung adeno-to-squamous transdifferentiation through remodeling tumor immune microenvironment
Source: Natl Sci Rev. 2023 Feb 14;10(4):nwad028. doi: 10.1093/nsr/nwad028 (PMC10084920; doi:10.1093/nsr/nwad028)
Supplement: nwad028_Supplemental_Files [file nwad028_supplemental_files.zip › supplmentary data.docx]

**SUPPLEMENTARY DATA**

**Counteracting lineage-specific transcription factor network finely tunes lung adeno-to-squamous transdifferentiation through remodeling tumor immune microenvironment**

Shijie Tang^1,#^, Yun Xue^1,2,#^ , Zhen Qin^1,#^, Zhaoyuan Fang^1,12,#^, Yihua Sun^3,4,#^, Chongzhe Yuan^3,4^, Yunjian Pan^3,4^, Yue Zhao^3,4^, Xinyuan Tong^1^, Jian Zhang^1^, Hsinyi Huang^1^, Yuting Chen^1,6^, Liang Hu^1^, Dasong Huang^5^, Ruiqi Wang^5^, Weiguo Zou^1^, Yuan Li^3,4^, Roman K Thomas^7^, Andrea Ventura^8^, Kwok-Kin Wong^9^, Haiquan Chen^3,4,*^, Luonan Chen^1,6,10*^, Hongbin Ji^1,6,11*^

**SUPPLEMENTARY METHODS**

**Somatic mutation calling**

WGS data were processed according to the Genome Analysis Toolkit (GATK, <https://gatk.broadinstitute.org>) [1]. First of all, raw fastq data were treated with Trimmomatic (v0.39) [2] for adapter trimming and low quality reads filtering and then aligned to hg38 human genome reference using BWA-mem (v0.7.15) [3]. Samtools (v1.4) [4] was used to convert the resulting SAM files to compressed BAM files and then sort the BAM files. PCR duplicates were marked with Picard, and base quality scores were recalibrated using BaseRecalibrator tool of GATK (v4.0.9.0). Next, Mutect2 [1] was run to call somatic mutations from the tumor-normal paired bam files. In addition, each normal file was conducted with tumor-only mode of Mutect2 and then created a panel of normal file to filter out expected artifacts and germline variations. The resulted VCF files were annotated with ANNOVAR [5]. MutsigCV (<https://software.broadinstitute.org/cancer/cga/>mutsig_run) was performed to identify genes with significant higher mutation rates than background mutation rates. COSMIC database and OncoKB database were used to define cancer related genes, and oncogenes and tumor suppressor genes were recorded in OncoKB database [6,7]. In addition, Cancer Genome Interpreter [8] was used to annotate whether the mutation was LOF (loss of function) or GOF (gain of function). As for the LCM sample analyses, we used Mutect2 and Strelka2 [9] to choose those variants with allele frequency higher than 0.05 in both methods for further analyses. For detecting *EGFR* mutations accurately in LCM samples, we combined Integrated Genomics Viewer (IGV) [10] results together with Mutect2 and Strelka2.

**Mutational signature analysis**

Somatic base substitutions were classified into 96 possible mutation trinucleotides and the somatic mutation rate of each type of substitutions was calculated to generate a context-specific mutation profile. Mutational signatures were extracted from the context-specific mutation profile by using non-negative matrix factorization algorithm (“MutationalPatterns” package) [11].

Pearson correlation coefficients (PCCs) between context-specific mutation profile of our data and mutational signatures in COSMIC database [7] were calculated, and signatures with PCC <0.4 in more than 90% samples were filtered out. Unsupervised hierarchical clustering was performed on PCC profile of 17 remained signatures (Signature 1, 2, 3, 4, 5, 6, 7, 8, 11, 13, 14, 16, 18, 19, 24, 29 and 30).

**Copy number analyses**

For WGS data, tumor-normal paired bam files were processed with CNVKit [12] to call somatic copy number variation and then GISTIC2.0 (Genomic Identification of Significant Targets in Cancer v2.0.23) [13] was used to identify focal gain and loss regions. CNV amplification and CNV deletion per gene referred to ‘2’ and ‘-2’ value in “all_thresholded.by_genes.txt” file generated from GISTIC2.0. CNV segments of LUAS samples, TCGA LUAD and TCGALUSC cohort were displayed using Integrated Genomics Viewer (IGV) [10]. CNV segments of LUAS were converted to hg19 coordinated by UCSC liftOver tool to unify the version of reference genome with TCGA cohorts. Percentage of samples with allele amplification were shown in red (log2(cn/2) >0.1) while percentage of samples with allele loss were shown in blue (log2(cn/2) <-0.1). Sequenza [14] was performed to get somatic copy number variation of LCM WGS data and circos plot was drawn with “all_thresholded.by_genes.txt” file generated from GISTIC2.0.

**DNA-based subtyping**

Integration subtyping of somatic mutations and copy number variations were conducted with a method combining Similarity Network Fusion (SNF) and Consensus clustering (R package “CancerSubtypes”) [15]. SNF was first applied to get the fusion patients similarity matrix which was then used as the sample distance for Consensus Clustering. Genes mutated in at least three samples (n=1131) or amplified/deleted in at least five samples (n=2735) were used.

**Gene fusion detection**

Three different algorithms including Fusioncatcher [16], Fusionmap [17] and Prada [18] were performed simultaneously to identify gene fusion events in RNA-seq data. Fusion events detected in at least two algorithms were chosen for further analyses.

**Cancer Cell Fraction**

PyClone [19] was used to estimate corrected cancer cell fraction (CCF) of somatic mutations in LCM samples in whole-genome scale and tumor purity was evaluated by ABSOLUTE [20]. Variants with total counts less than 15 were filtered to improve the algorithm reliability and speed. The CCFs of mutations were plotted between paired samples in the same patient. CCF plots were used to deduce the potential progression routes between adenomatous and squamous components. Theoretically, progression is usually initiated by subclones with CCF less than 1 and these mutations will be inherited by later lesion with CCF close to 1 during the transition process [21,22].

**RNA-seq data analyses**

RNA-seq data were aligned to hg38 human genome reference using STAR (v2.6.0) [23]. Depth correction and log2 normalization were performed on raw count data. Genes expressed as zero in more than 85% samples were filtered out. Multidimensional scaling plot was processed with plotMDS function in limma package [24]. To comparatively analyze the gene expression, RNA Seq data of TCGA LUAD (tumor: n=526, normal: n=59) and TCGA LUSC (tumor: n=550, normal: n=49) were downloaded from UCSC Xena (<https://xenabrowser.net>), from which samples with tumor purity > 0.5 were used [25] (LUAD: n=430, LUSC: n=433). RNA Seq data of Asian LUAD was downloaded from cBioPortal (https://www.cbioportal.org/). Combat function in SVA package was used to correct the batch effects [26]. “GSVA” package [27] was used to calculate the expression scores of different immune cell types.

**mRNA-based Subtyping**

Consensus clustering (‘‘ConsensusClusterPlus’’ package; clusterAlg= "hc", distance= "pearson", innerLinkage= "ward.D2") [28] was performed on gene expression profile of 4100 genes with high variance across samples (standard deviation in top 20%). Signature genes in each mRNA-based subtype were calculated using the Student’s t test and adjusted using “BH” method [29] (Cutoff: FDR < 0.01, Foldchange ≥ 2.5). Gene Set Enrichment Analysis (GSEA) was performed using the GSEA software (v4.0.3) [30] on mRNA expression data to get enriched KEGG pathways in each mRNA-based subtype. To compare mRNA subtypes with known TCGA LUAD or LUSC subtypes, we have calculated the average Euclidean distance between each LUAS-subtype and LUAD/LUSC subtype to measure their similarities.

**Gene expression differential analysis and upstream analysis**

Gene expression differential analysis between TRU-like subtype and basal-like subtype were performed using Student’s t test and adjusted using “BH” method [29]. Genes with FDR < 0.05 and Fold Changes ≥ 1.5 were regarded as differentially expressed genes (DEGs). Upstream transcription factors (TFs) of these DEGs were inferred using Ingenuity Pathway Analysis (IPA)[32] and TFs with FDR< 0.05, absolute log2FoldChange ≥ 1.5 and absolute activated Z-score ≥ 1 were regarded as top upstream TFs. Regulatory networks were then plotted using IPA together with top upstream TFs and directly linked DEGs [32].

**RNA-seq data and dynamic network biomarker (DNB) analysis**

LUAD GSVA score were calculated using the top 100 up-regulated genes between TCGA LUAD and TCGA LUSC cohort whereas LUSC GSVA score were calculated using the top 100 up-regulated genes between TCGA LUSC and TCGA LUAD cohort [27]. Human LUAS samples were then ranked from the highest LUAD GSVA score to the highest LUSC GSVA score.

Window-scaling dynamic network biomarker analysis [33-37] was performed on RNA-seq data of these ordered samples, and 10 samples per window were used for sliding-window to reduce the potential noise (total gene number=20501). It has been shown that when the biological system approaches the critical state or tipping point during a dynamical process, expression of DNB genes simultaneously satisfies three generic properties: (a) the average Pearson's correlation coefficient (PCC_in_) of DNB genes as a group drastically increases; (b) The average Pearson's correlation coefficient (PCC_out_) of DNB genes between this group and any others drastically decreases; (c) The average standard deviation (SD_in_) of DNB genes in this group drastically increases. These three generic properties could be combined together to construct a DNB composite index (CI)

$CI={SD}_{in}\frac{{PCC}_{in}}{{PCC}_{out}}$  _(S1)_

to estimate the critical state or tipping point during a dynamical process based on the observed RNA-seq data of each sliding window (each 10 samples), i.e., when the CI at a sliding window reaches the highest value, the state of the ten samples at this sliding window is considered as the critical state/tipping point. The corresponding DNB genes are considered as the leading molecules of this critical transition.

**Bifurcation and potential landscape analysis of the four-TF regulatory network**

To qualitatively explore the dynamical features of human LUAS development further, a mathematical model of the four-TF regulatory network (Figure 4F), i.e., *FOXA2* (*F)*, *NKX2-1* (*N)*, *TP63* (*T)*, and *SOX2* (*S*) by four ordinary differential equations (ODEs) with the Hill functions in terms of their concentrations is given as follows:

$$\dot{F}=\frac{\alpha_{F}}{1+a_{1}T^{2}+a_{2}S^{2}}-d_{F}F,$$

$$\dot{N}=\alpha_{N}\frac{1+F^{2}}{1+a_{3}F^{2}+a_{4}S^{2}}-d_{N}N,$$

$$\dot{T}=\alpha_{T}\frac{1+S^{2}}{1+a_{5}S^{2}+a_{6}N^{2}}-d_{T}T,$$

$\dot{S}=\beta_{S}\frac{S^{2}}{a_{7}^{2}+S^{2}}+\frac{\alpha_{S}}{1+a_{8}F^{2}+a_{9}N^{2}}-d_{S}S.$ (S2)

The definition of each parameter and its basal value was listed in Table S6 with normalized values. Clearly, Eqn.(S2) is a nonlinear dynamical system with four variables (*F,N,T,S*).

The steady states of the model can be numerically solved and plotted as a function of some specified parameters, e.g., $\alpha_{S}$ is for bifurcation analysis of the four-TF network. When $\alpha_{S}$ is low, the system stays mainly at the adenomatous state. As $\alpha_{S}$ increases until it reaches a critical value of 0.18 which is the tipping point (considered as bi-stability with both adenomatous and squamous states). As $\alpha_{S}$continues to increase, the squamous state becomes dominant (Figure S5I). The bifurcation diagrams for all four TFs against the parameter $\alpha_{S}$ were given in Figure S5I.

The next step is to display the development process from the viewpoint of a potential energy landscape [38]. After four ODEs are constructed, we further considered the corresponding stochastic dynamics. The steady-state probability distribution $P_{ss}(F,N,T,S)$ of the four variables can be obtained by stochastic simulation [38,39]. Thus, we can acquire the potential landscape $U$ of the system as

$U=-ln(P_{ss})$ (S3)

where $P_{ss}$ represents the probability distribution of the steady state (or cell state probability) and $U$ the dimensionless potential energy. The lowest potential $U$ means the highest probability of cell state while the highest potential $U$ implies the lowest probability of cell state.

The potential landscape along the two-dimensional *SOX2*-*FOXA2* space is calculated. Figure 4I shows three-dimensional landscapes for the system in terms of *FOXA2* and *SOX2* as an illustrative example (we can have the similar landscape using other variables). Clearly, there are two states on the landscape (bi-stability, Figure 4I). Potential landscape or *U* reflects the steady-state probability distribution of cell population described by the four-TF network. The three landscapes in Figure 4I were obtained by $\alpha_{S}$=0.1, 0.3, 0.8 (left, middle, right), respectively. The change of regulation strength mimicking the micro-environment change or system rewiring, e.g., $\alpha_{S}$ refers to the change of landscape topography or state.

**Cell culture, CRISPR Lentivirus preparation**

*Kras^G12D/+^* mouse embryonic fibroblasts (MEFs), *Kras^G12D^/Trp53^-/-^* (KP) and HEK-293T cells were cultured in DMEM supplemented with 8% FBS. HEK-293T cells were obtained from the American Type Culture Collection. MEF was generated from *Kras^G12D/+^* mice [40]. KP cells were generated from *Kras^G12D/+^;Trp53^fl/fl^* mice [41]. All cell lines were free of mycoplasma contamination.

Lentiviruses were produced by transfection of HEK-293T cells with pSECC or LentiCRISPRv2 constructs and packaging vectors (psPAX2 and pMD2.G). For pSECC lentiviruses, supernatant was collected 48 hours post-transfection, concentrated by ultracentrifugation at 50,000g for 2 hours and resuspended overnight in an appropriate volume of OptiMEM (Gibco).

**Western Blotting**

Cell lysates were prepared and performed western blotting analysis with the following antibodies: FOXA2 antibody (ab108422; Abcam), SOX2 antibody (A0561; Abclonal) and β-actin (AC026; Abclonal).

**Hematoxylin-eosin staining, immunohistochemistry and immunostaining.**

Hematoxylin-eosin (HE) staining, immunohistochemistry (IHC) and immunostaining were performed as previously described [42]. The following antibodies were used: p63 (ab124762; Abcam), SOX2 (ab92494; Abcam), KRT5 (BS1208; Bioworld), TTF1 (ab133638; Abcam), FOXA2 (ab108422; Abcam), MPO (AF3667; R&D system), DAPI (#564907; BD Bioscience).

**REFERENCES**

1. McKenna, A., et al., *The Genome Analysis Toolkit: a MapReduce framework for analyzing next-generation DNA sequencing data.* Genome research, 2010. **20**(9): p. 1297-1303.

2. Bolger, A., M. Lohse, and B. Usadel, *Trimmomatic: a flexible trimmer for Illumina sequence data.* Bioinformatics, 2014. **30**(15): p. 2114-2120.

3. Li, H., *Aligning sequence reads, clone sequences and assembly contigs with BWA-MEM.* arXiv: Genomics, 2013.

4. Li, H., et al., *The Sequence Alignment/Map format and SAMtools.* Bioinformatics, 2009. **25**(16): p. 2078-2079.

5. Wang, K., M. Li, and H. Hakonarson, *ANNOVAR: functional annotation of genetic variants from high-throughput sequencing data.* Nucleic acids research, 2010. **38**(16): p. e164-e164.

6. Chakravarty, D., et al., *OncoKB: a precision oncology knowledge base.* JCO precision oncology, 2017. **1**: p. 1-16.

7. Tate, J.G., et al., *COSMIC: the Catalogue Of Somatic Mutations In Cancer.* Nucleic Acids Research, 2018. **47**(D1): p. D941-D947.

8. Tamborero, D., et al., *Cancer Genome Interpreter annotates the biological and clinical relevance of tumor alterations.* Genome Medicine, 2018. **10**(1): p. 25.

9. Kim, S., et al., *Strelka2: fast and accurate calling of germline and somatic variants.* Nature Methods, 2018. **15**(8): p. 591-594.

10. Robinson, J.T., et al., *Integrative genomics viewer.* Nature biotechnology, 2011. **29**(1): p. 24.

11. Blokzijl, F., et al., *MutationalPatterns: comprehensive genome-wide analysis of mutational processes.* Genome medicine, 2018. **10**(1): p. 33.

12. Talevich, E., et al., *CNVkit: genome-wide copy number detection and visualization from targeted DNA sequencing.* PLoS computational biology, 2016. **12**(4): p. e1004873.

13. Mermel, C.H., et al., *GISTIC2. 0 facilitates sensitive and confident localization of the targets of focal somatic copy-number alteration in human cancers.* Genome biology, 2011. **12**(4): p. R41.

14. Favero, F., ., et al., *Sequenza: allele-specific copy number and mutation profiles from tumor sequencing data.* Annals of Oncology Official Journal of the European Society for Medical Oncology, 2015. **26**(1): p. 64.

15. Xu, T., et al., *CancerSubtypes: an R/Bioconductor package for molecular cancer subtype identification, validation and visualization.* Bioinformatics, 2017. **33**(19): p. 3131-3133.

16. Nicorici, D., et al., *FusionCatcher - a tool for finding somatic fusion genes in paired-end RNA-sequencing data.* bioRxiv, 2014: p. 011650.

17. Ge, H., et al., *FusionMap: detecting fusion genes from next-generation sequencing data at base-pair resolution.* Bioinformatics, 2011. **27**(14): p. 1922-1928.

18. Wandaliz, T.G., et al., *PRADA: pipeline for RNA sequencing data analysis.* Bioinformatics, 2014. **30**(15): p. 2224.

19. Roth, A., et al., *PyClone: statistical inference of clonal population structure in cancer.* Nature methods, 2014. **11**(4): p. 396.

20. Carter, S.L., et al., *Absolute quantification of somatic DNA alterations in human cancer.* Nat Biotechnol, 2012. **30**(5): p. 413-21.

21. Xue, R., et al., *Genomic and Transcriptomic Profiling of Combined Hepatocellular and Intrahepatic Cholangiocarcinoma Reveals Distinct Molecular Subtypes.* Cancer Cell, 2019. **35**(6): p. 932.

22. Gundem, G., et al., *The evolutionary history of lethal metastatic prostate cancer.* Nature, 2015. **520**(7547): p. 353-357.

23. Dobin, A., et al., *STAR: ultrafast universal RNA-seq aligner.* Bioinformatics, 2013. **29**(1): p. 15-21.

24. Ritchie, M.E., et al., *limma powers differential expression analyses for RNA-sequencing and microarray studies.* Nucleic acids research, 2015. **43**(7): p. e47-e47.

25. Aran, D., M. Sirota, and A.J. Butte, *Systematic pan-cancer analysis of tumour purity.* Nature communications, 2015. **6**: p. 8971.

26. Leek, J.T., et al., *The sva package for removing batch effects and other unwanted variation in high-throughput experiments.* Bioinformatics, 2012. **28**(6): p. 882-883.

27. Hänzelmann, S., R. Castelo, and J. Guinney, *GSVA: gene set variation analysis for microarray and RNA-seq data.* BMC bioinformatics, 2013. **14**(1): p. 7.

28. Wilkerson, M.D. and D.N. Hayes, *ConsensusClusterPlus: a class discovery tool with confidence assessments and item tracking.* Bioinformatics, 2010. **26**(12): p. 1572-1573.

29. Benjamini, Y. and Y. Hochberg, *Controlling the false discovery rate: a practical and powerful approach to multiple testing.* Journal of the Royal statistical society: series B (Methodological), 1995. **57**(1): p. 289-300.

30. Subramanian, A., et al., *Gene set enrichment analysis: a knowledge-based approach for interpreting genome-wide expression profiles.* Proceedings of the National Academy of Sciences, 2005. **102**(43): p. 15545-15550.

31. Hoshida, Y., *Nearest template prediction: a single-sample-based flexible class prediction with confidence assessment.* PLoS One, 2010. **5**(11): p. e15543.

32. Krämer, A., et al., *Causal analysis approaches in ingenuity pathway analysis.* Bioinformatics, 2014. **30**(4): p. 523-530.

33. Chen, L., et al., *Detecting early-warning signals for sudden deterioration of complex diseases by dynamical network biomarkers.* Scientific Reports, 2012. **2**(1): p. 342-342.

34. Yang, B., et al., *Dynamic network biomarker indicates pulmonary metastasis at the tipping point of hepatocellular carcinoma.* Nature Communications, 2018. **9**(1): p. 678-678.

35. Liu, X., et al., *Detection for disease tipping points by landscape dynamic network biomarkers.* National Science Review, 2019. **6**(4): p. 775-785.

36. Liu, X., et al., *Quantifying critical states of complex diseases using single-sample dynamic network biomarkers.* PLOS Computational Biology, 2017. **13**(7).

37. Lesterhuis, W.J., et al., *Dynamic versus static biomarkers in cancer immune checkpoint blockade: unravelling complexity.* Nature Reviews Drug Discovery, 2017. **16**(4): p. 264-272.

38. Wang, J., et al., *Quantifying the Waddington landscape and biological paths for development and differentiation.* Proceedings of the National Academy of Sciences of the United States of America, 2011. **108**(20): p. 8257-8262.

39. Shi, J., et al., *Energy landscape decomposition for cell differentiation with proliferation effect.* National Science Review, 2022. **9**(8).

40. Wang, Z.G., et al., *Temporal Dissection of K-ras G12D Mutant In Vitro and In Vivo Using a Regulatable K-ras G12D Mouse Allele.* PLOS ONE, 2012. **7**(5).

41. Wu, Q., et al., *In vivo CRISPR screening unveils histone demethylase UTX as an important epigenetic regulator in lung tumorigenesis.* Proceedings of the National Academy of Sciences of the United States of America, 2018. **115**(17): p. 201716589.

42. Li, F., et al., *LKB1 inactivation elicits a redox imbalance to modulate non-small cell lung cancer plasticity and therapeutic response.* Cancer cell, 2015. **27**(5): p. 698-711.

**SUPPLEMENTARY FIGURES**


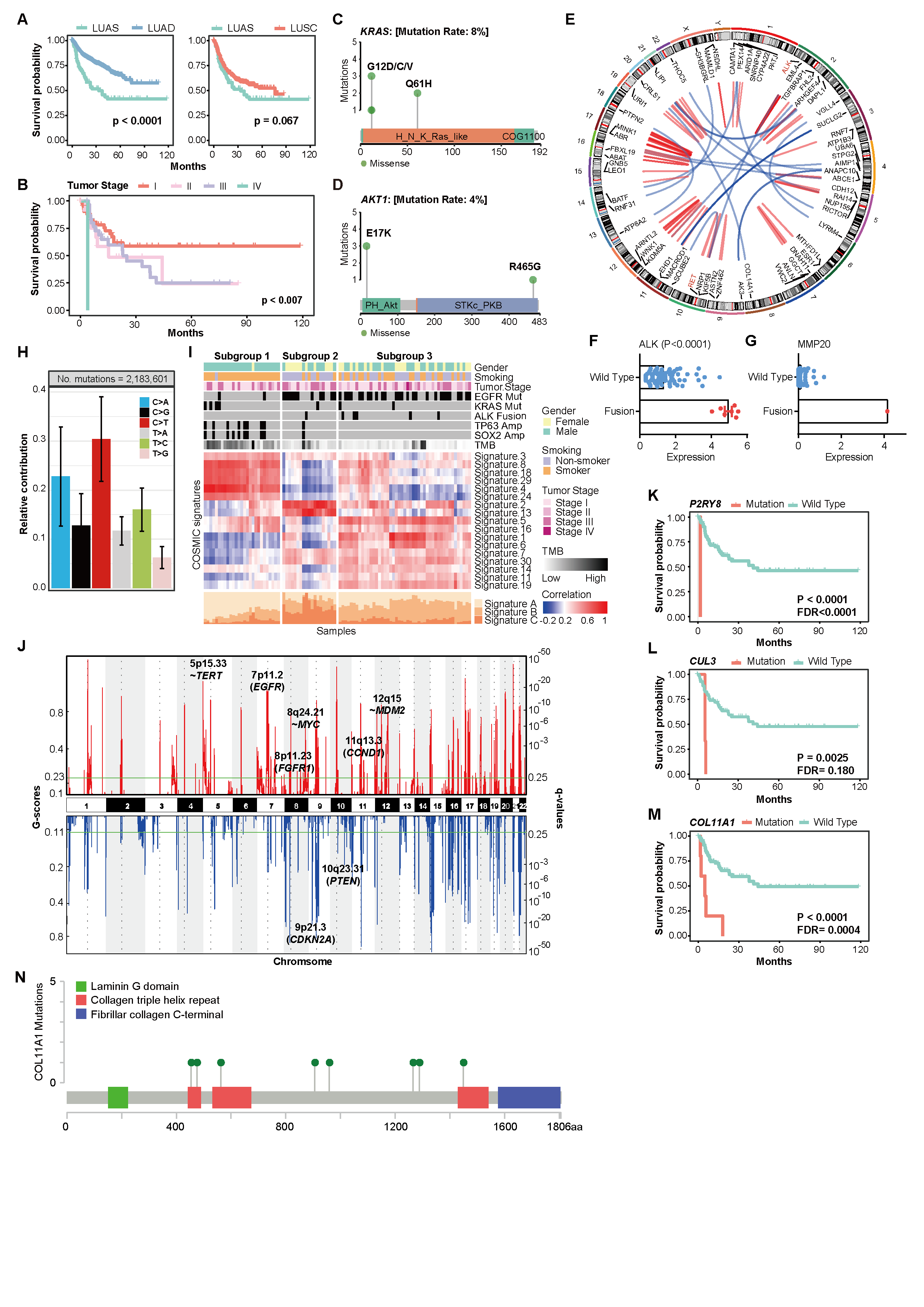


**Figure S1, Related to Figure 1**

**A**, Kaplan-Meier survival curves for relapse-free survival (RFS) of LUAS patients (n=120) in comparison with LUAD patients (n=3295) and LUSC patients (n=893) from Chinese NSCLC samples collected during the period from 2007 to 2017. **B**, Kaplan-Meier survival curves for relapse-free survival (RFS) of 109 Chinese LUAS patients with indicated pathological stages. **C-D**, Hotspot mutations of *KRAS* (C) and *AKT1* (D). **E**, Graphical representation of in-frame fusion events identified from 93 human LUAS transcriptome data. Intra-chromosome fusions were indicated with red lines joining two genomic loci, whereas inter-chromosome fusions were indicated with blue lines. **F-G**, Expression of ALK (F) and MMP20 (G) in fusion events compared to wild type. P-value was calculated with Student’s t-test. **H**, Bar plot showing the relative contribution of different types of genome-wide point mutations. **I**, Heatmap of mutational signature-based clustering. Pearson correlation coefficients (PCCs) between context-specific mutation profile of LUAS and mutational signatures in COSMIC database were shown. Each row indicated a COSMIC signature, and each column indicated a sample. Clinical features, DNA alterations and TMB were displayed on top, whereas relative contribution of three *de novo* signatures was shown on bottom. **J**, GISTIC plots of significant focal amplification (red) and deletion (blue) regions with G-scores (left) and q-values (right). **K-M**, Kaplan-Meier survival curves for relapse-free survival (RFS) of patients with *CUL3* mutations (**K, n=4**), *P2RY8* mutations (**L, n=3**) and *COL11A1* mutations (**M, n=8**). Log-rank test was used for statistical analyses. The false discovery rate (FDR) was used to correct the results of multiple comparisons. **N**, Lollipop plot showing mutations of COL11A1 in Chinese LUAS.


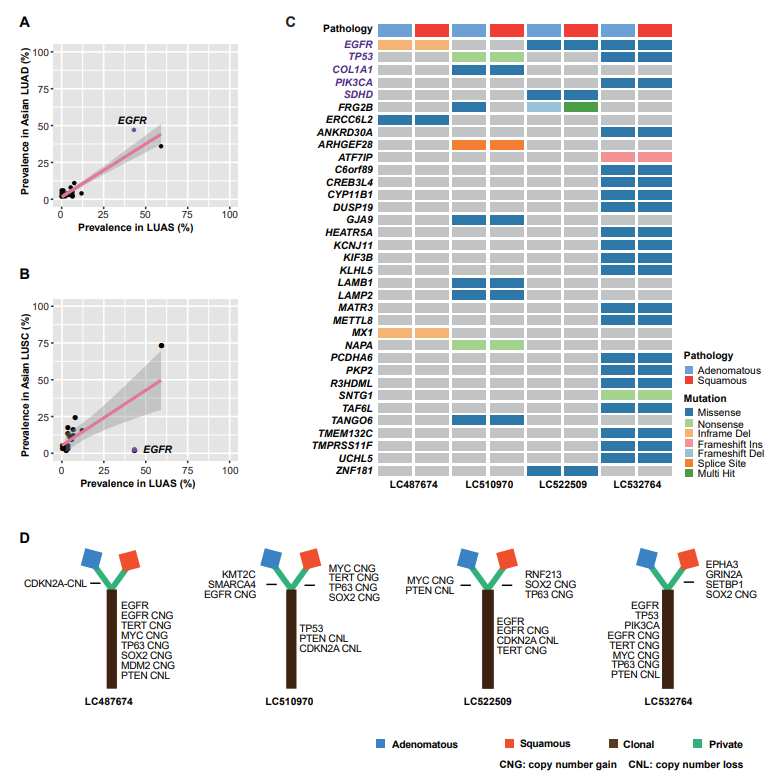


**Figure S2, Related to Figure 2**

**A**, Consistency plot between the prevalence of top mutations in Asian LUAD and Chinese LUAS. The line was fitted with linear regression. **B**, Consistency plot between the prevalence of top mutations in Asian LUSC and Chinese LUAS. The line was fitted with linear regression. R-squared score and Pearson correlation coefficient were indicated. **C**, Somatic mutation plot of all top mutations (frequency ≥ 25%) in 4 paired adenomatous and squamous components. Cancer-related genes reported in OncoKB and COSMIC database were in purple. **D**, Phylogenetic trees of 4 paired LCM samples showed the clonal origin and branch evolution of LUAS. Top cancer-related mutations and CNVs were shown. Length of the trunk or branch was not correlated to the number of variations.


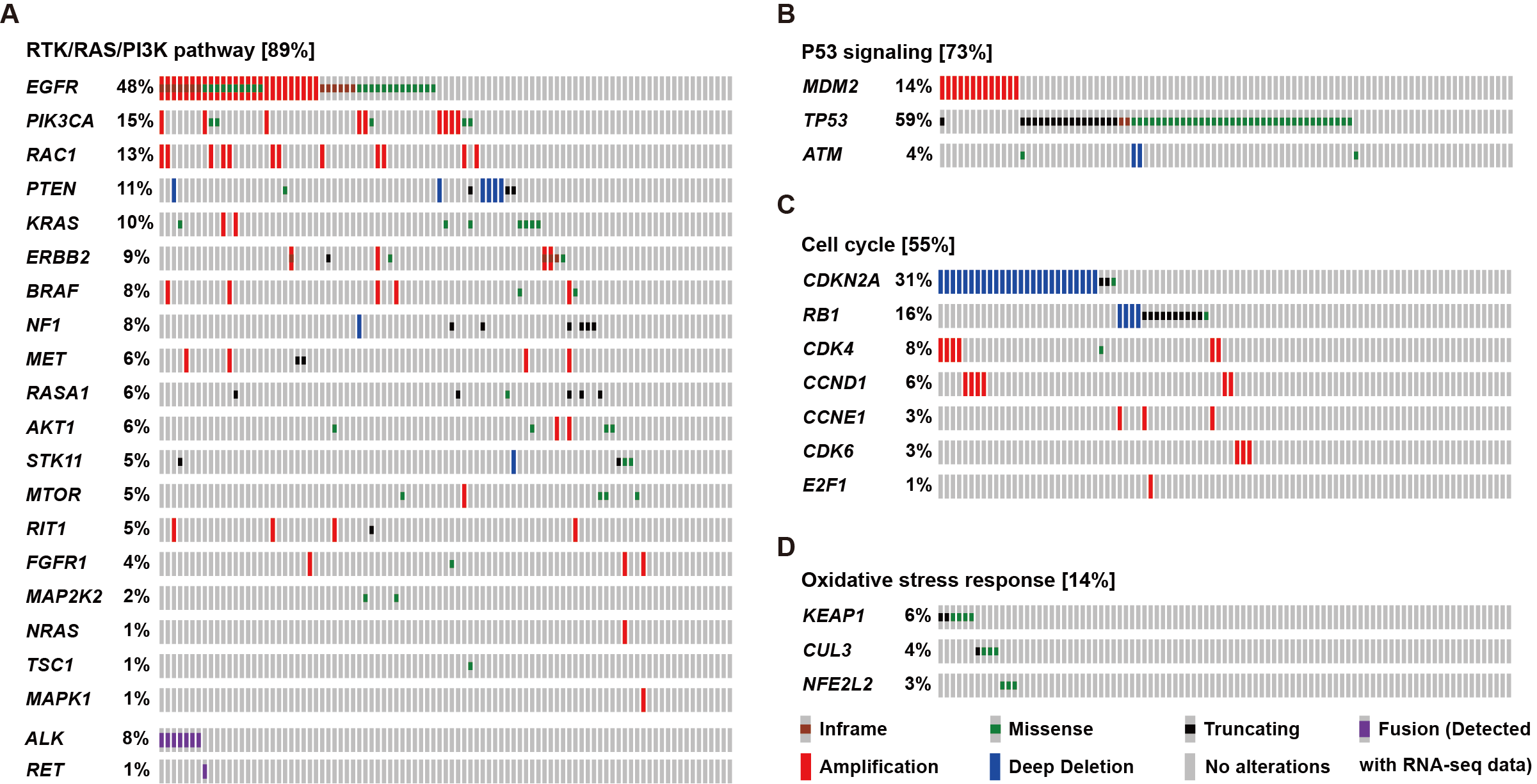


**Figure S3, Related to Figure 3**

**A-D**, Detailed genetic alterations of RTK/RAS/PI3K pathway (**A**), P53 signaling (**B**), Cell cycle (**C**) and Oxidative stress response (**D**) in Chinese LUAS.

**
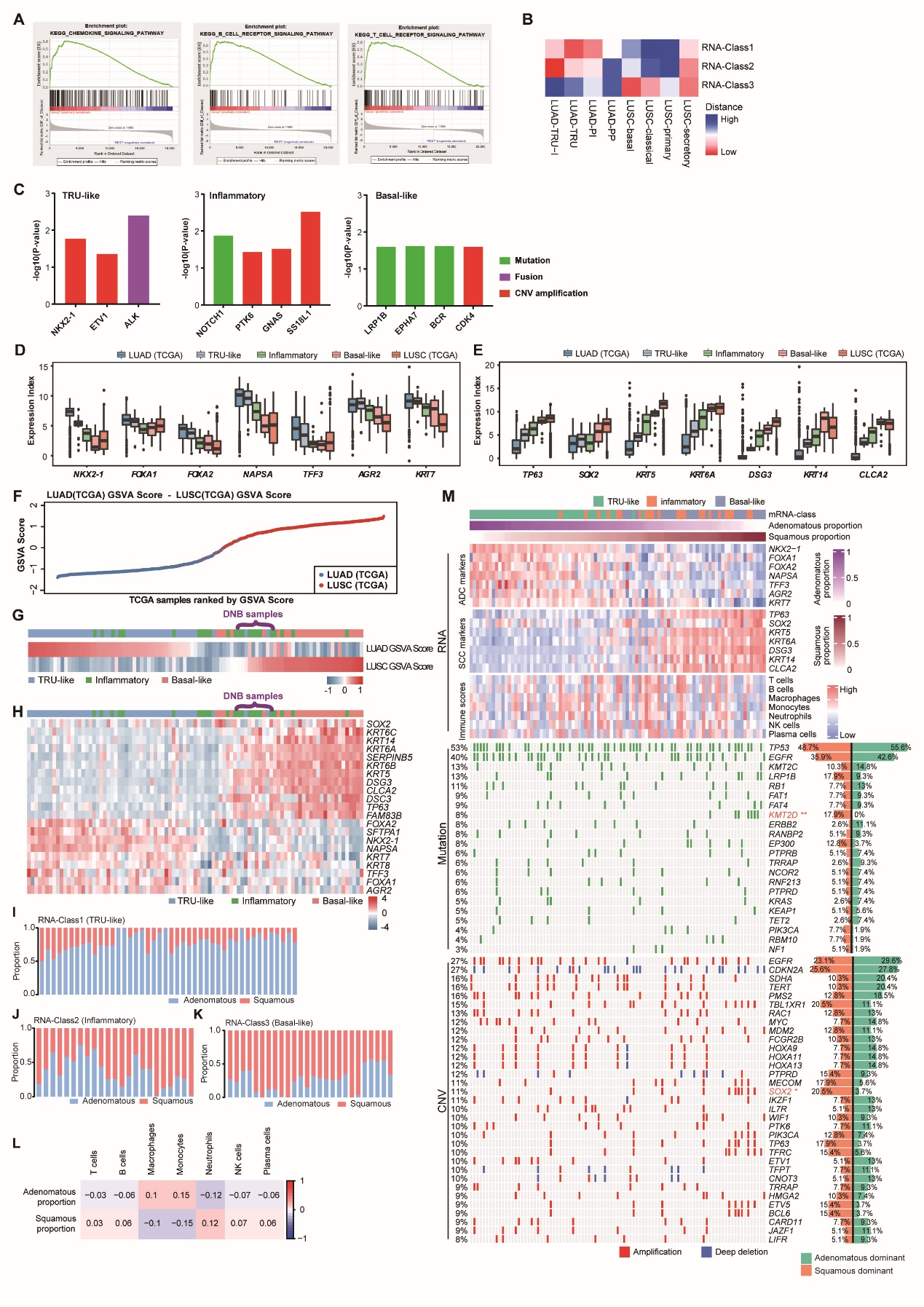
**

**Figure S4, Related to Figure 4**

**A**, GSEA plot of three pathways enriched in the inflammatory subtype. **B**, Heatmap showing average Euclidean distance among LUAS, LUAD and LUSC subtypes. TRU, PI and PP are subtypes of TCGA LUAD while TRU-I was subtype of Asian LUAD. Basal, classical, primary and secretory are subtypes of TCGA LUSC. (TRU: terminal respiratory unit; PI: proximal inflammatory; PP: proximal proliferative; TRU-I: TRU-inflammatory) **C**, Enriched genomic alterations among three indicated classes. *ALK* fusions were calculated using the RNA-seq data from 93 LUAS whereas other genomic alterations were calculated using both WGS and RNA-seq data from 81 LUAS. Fisher’s exact test. **D-E**, Comparison of GSVA scores for adenomatous biomarkers (**D**), squamous biomarkers (**E**) in three different LUAS classes together with TCGA LUAD and LUSC. **F**, The TCGA cancer samples were aligned from LUAD to LUSC based on the GSVA score. **G**, LUAS samples were ranked from the highest LUAD GSVA score to the highest LUSC GSVA score. DNB samples were indicated in the context with various classes of LUAS. **H**, Heatmap of known LUAD and LUSC markers’ gene expression in GSVA score-ranked LUAS. **I-K,** Proportion of adenomatous and squamous components in three RNA-based subtypes TRU-like (**I**), Inflammatory (**J**) and Basal-like (**K**) deduced by the deconvolution of RNA expression matrix based on non-negative matrix factorization (NMF). **L**, Pearson correlation analysis between adenomatous/squamous proportions and GSVA scores for seven immune cell types. **M**, LUAS samples were ranked based on the decrease of adenomatous-to-squamous (A-to-S) ratio. Expression of known LUAD and LUSC markers, and GSVA scores of seven immune cell types were shown on the top. Alteration plot of top mutated and amplified/deleted cancer-related genes were shown on the bottom. Right bar graph indicated the rates of individual gene alteration in samples with adenomatous dominant (A-to-S >1) or squamous dominant (A-to-S <1). Genes with significant differences between these two groups were highlighted in red (*: P<0.05; ** P<0.01, Fisher’s exact test).

**
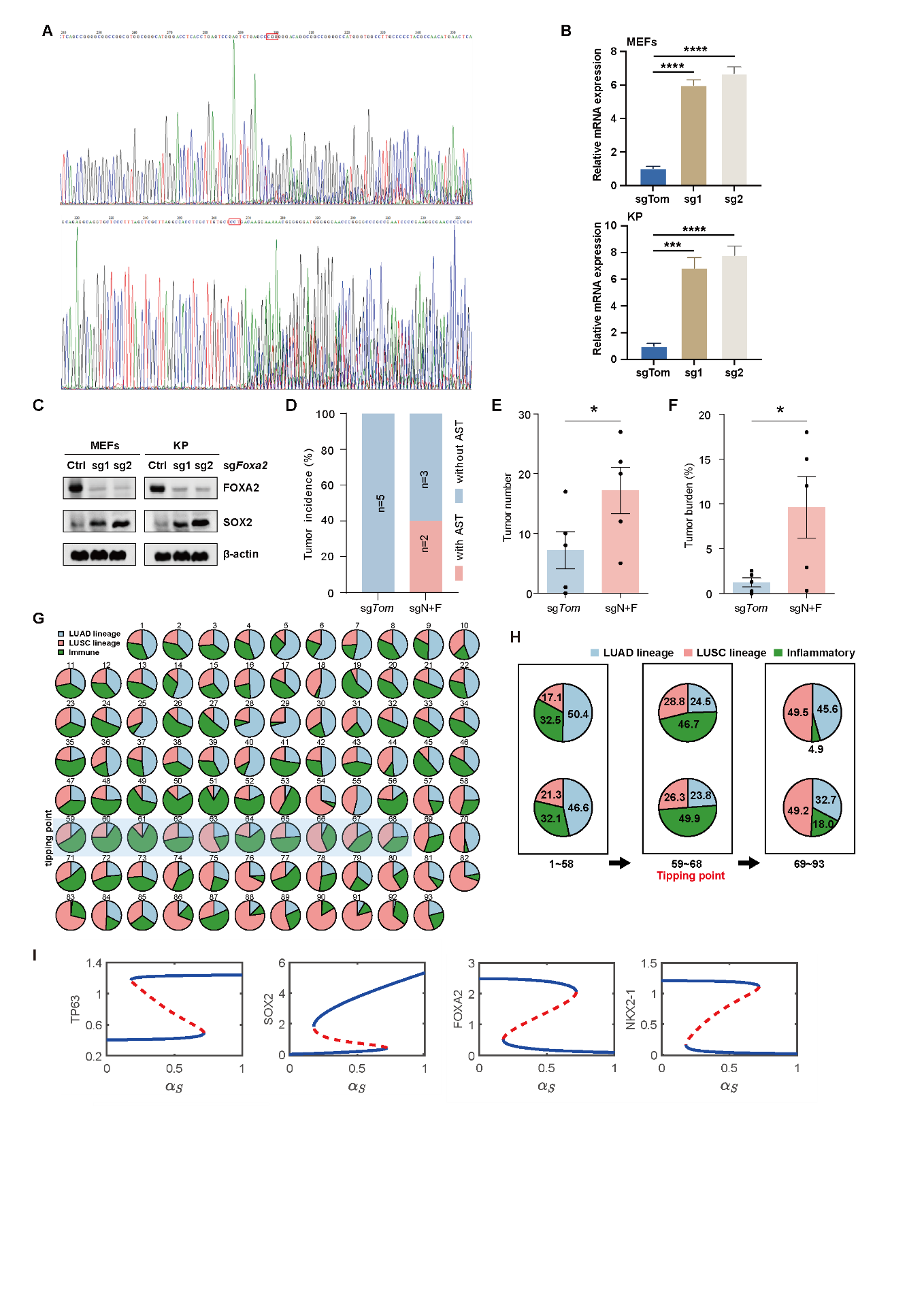
**

**Figure S5, Related to Figure 4**

**A**, Knockout efficiency detection of sg*Foxa2* and the proper edition were confirmed in targeted alleles by Sanger sequence. The ‘NGG’ site is marked by red box. **B**, Real-time PCR quantification of *Sox2* mRNA levels in MEFs and KP mouse lung cancer cell lines with *Foxa2* knockout. Student’s t-test, *P < 0.05, **P < 0.01, ***P < 0.001. **C**, Western blots detecting the levels of FOXA2 and SOX2 in *Kras^G12D/+^* mouse embryonic fibroblast (MEFs) and *Kras^G12D^/Trp53^-/-^* (KP) lung cancer cell line with or without *Foxa2* knockout. **D-F**, Quantification of tumor incidence (D) tumor number (E) and tumor burden (F) in *Kras^G12D/+^*; *Rosa26^LSL-Cas9^* mice treated with sg*Tom* and sgN+F. Data were shown as mean ± SEM. *P < 0.05, **P < 0.01. **G**, ­­­Pie charts showing the proportion of expression of LUAD/LUSC lineage TFs and GSVA score of immune genes in the ranked LUAS. The average gene expression of LUAD/LUSC lineage TFs and the GSVA score of immune genes were scaled by min-max normalization (range: 0-1). **H**, Pie charts of typical samples before/at/after the tipping point with proportional expression of LUAD/LUSC lineage TFs and GSVA score of immune genes. The average gene expression of LUAD/LUSC lineage TFs and the GSVA score of immune genes were scaled by min-max normalization (range: 0-1). **I**, Bifurcation diagrams with $\alpha_{S}$ as a control parameter for the four-TF regulatory network as a nonlinear dynamical system (Eqn.(S2) in Supplementary Information), and the y-axis represents concentrations of the four TFs (TP63, SOX2, FOXA2 and NKX2-1) or variables of the dynamical system.

**
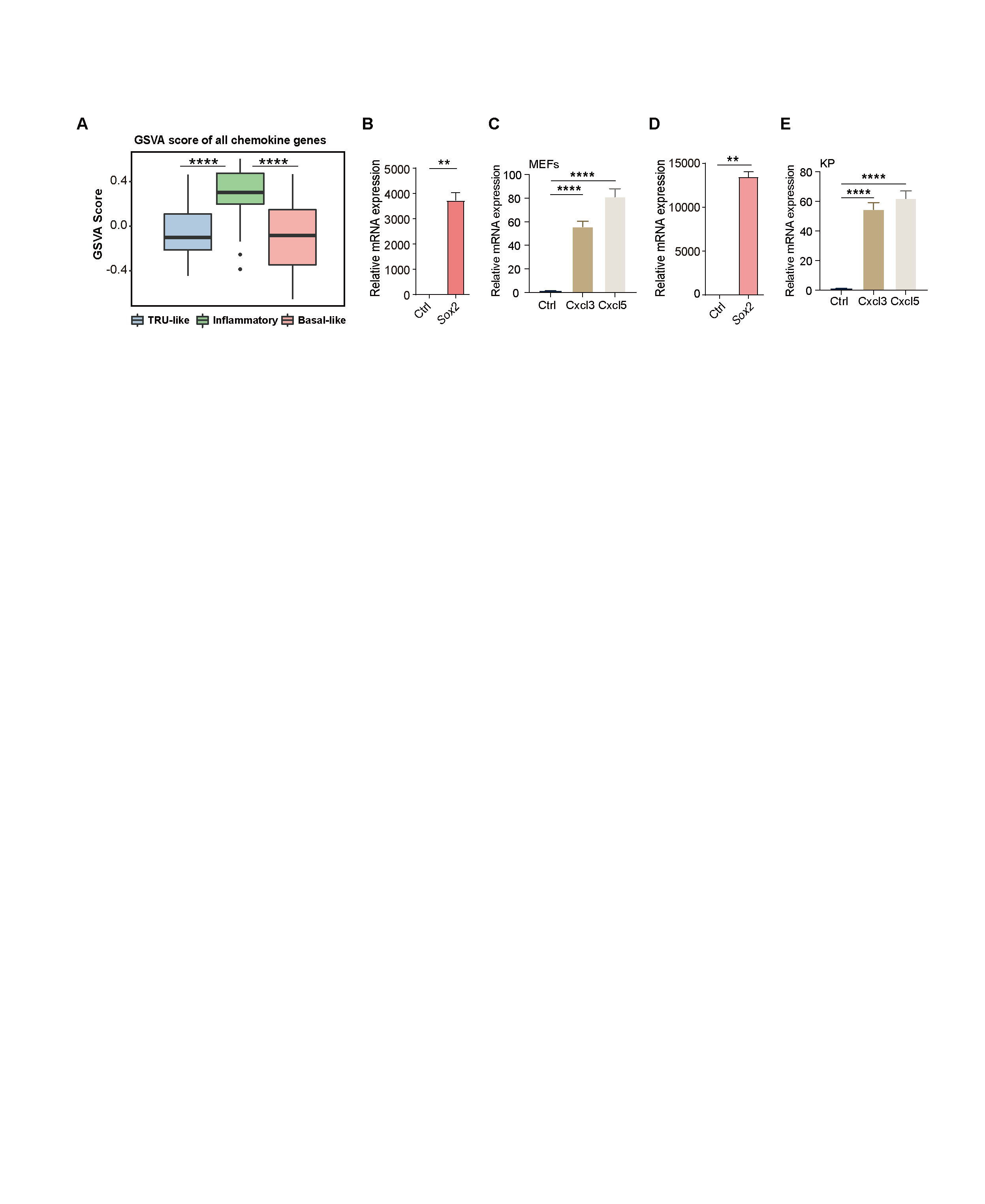
**

**Figure S6, Related to Figure 5**

**A**, GSVA score of all chemokines and their receptors among three different LUAS classes. Student’s t-test, ****P < 0.0001. **B-C**, Real-time PCR quantification of *Sox2*, *Cxcl3* and *Cxcl5* mRNA levels in KP mouse lung cancer cell line with *Sox2* overexpression. Student’s t-test, **P < 0.01. **D-E**, Real-time PCR quantification of *Sox2, Cxcl3* and *Cxcl5* mRNA levels in MEFs with *Sox2* overexpression. Student’s t-test, **P < 0.01.

**
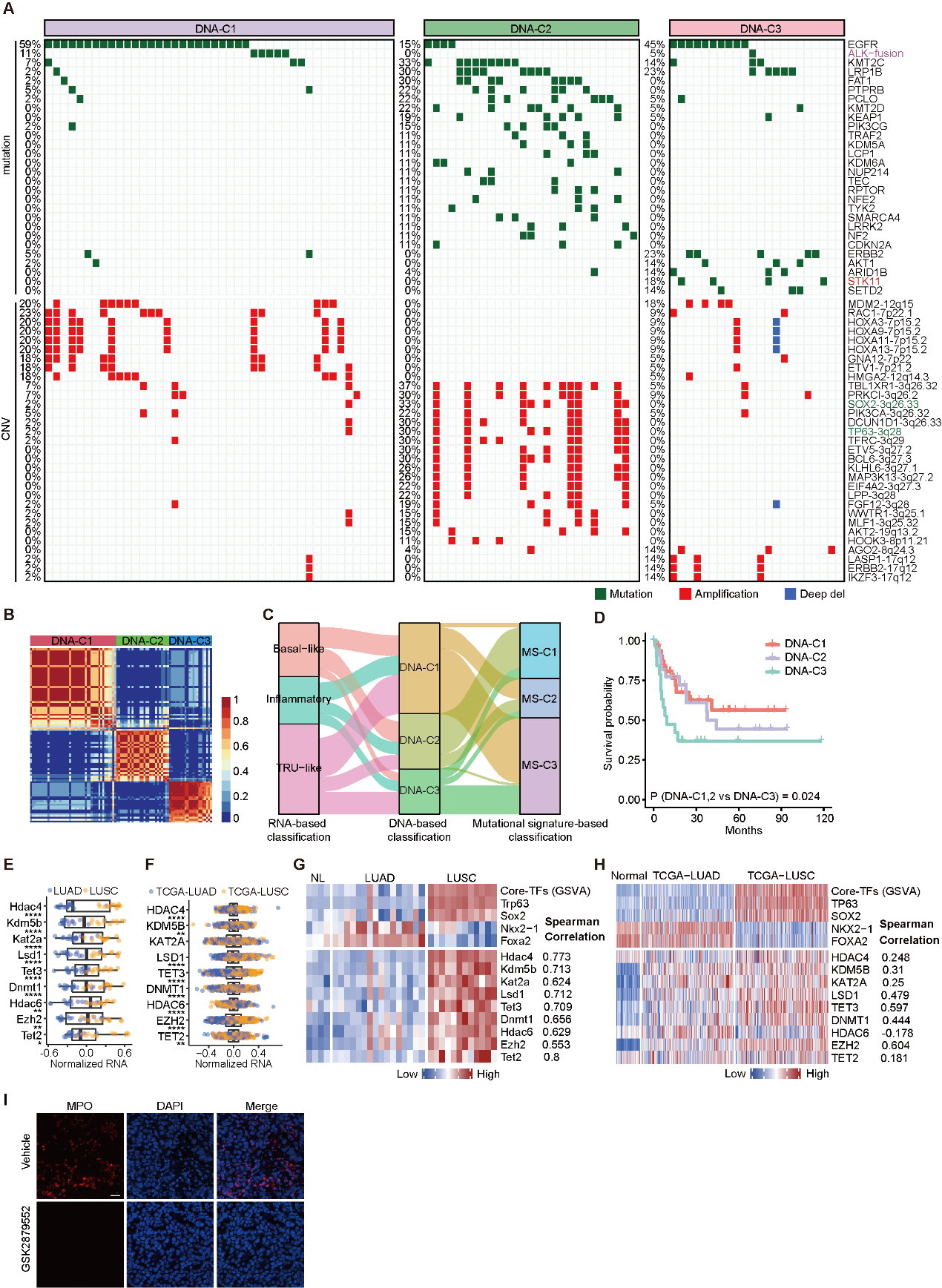
**

**Figure S7, Related to Figure 6**

**A**, Significantly enriched genomic alterations among three DNA classes (Fisher’s exact test). Chromosome band information of the CNVs was also listed. **B**, Heatmap showing similarity matrix of integration subtyping. **C,** Sankey diagram showing the relationship among transcriptome subgroups TRU-like, inflammatory, Basal-like, DNA subgroups C1-C3 and mutational signature-based subgroups C1-C3. **D**, Kaplan-Meier survival curves of relapse-free survival (RFS) in three DNA classes. P-value was calculated between class 3 and the remaining two classes. **E**, Boxplot showing differentially expressed epigenetic factors in LUSC versus LUAD in KL mouse RNA-seq data (FDR<0.05; Fold change>=1.5, ****:FDR<0.0001; ***:FDR<0.001; **FDR<0.01; *:FDR<0.05). **F**, Boxplot showing differences of epigenetic factors (shown in E) in TCGA LUSC versus TCGA LUAD (****:FDR<0.0001; ***:FDR<0.001; **FDR<0.01; *:FDR<0.05). **G**, Heatmap showing the expression of deregulated epigenetic factors (shown in E) in mouse KL tumors and their spearman correlation with GSVA scores of core-TFs (defining with GSVA scores of two LUAD lineage TFs minus those of two LUSC lineage TFs, right). **H**, Heatmap showing the expression of deregulated epigenetic factors (shown in E) and their spearman correlation with GSVA scores of core-TFs in TCGA datasets. I, Representative fluorescence staining in KL mice treated with vehicle or GSK2879552. Red: myeloperoxidase (MPO); blue: DAPI. Scale bar: 20µm.
